# Ror2 signaling regulated by differential Wnt proteins determines pathological fate of muscle mesenchymal progenitors

**DOI:** 10.1038/s41419-024-07173-9

**Published:** 2024-10-29

**Authors:** Koki Kamizaki, Mitsuko Katsukawa, Ayano Yamamoto, So-ichiro Fukada, Akiyoshi Uezumi, Mitsuharu Endo, Yasuhiro Minami

**Affiliations:** 1 Division of Cell Physiology, Department of Physiology and Cell Biology, Graduate School of Medicine, Kobe University, Kobe, Japan; 2 Laboratory of Stem Cell Regeneration and Adaptation, Graduate School of Pharmaceutical Sciences, Osaka University, Osaka, Japan; 3 Division of Cell Heterogeneity, Medical Institute of Bioregulation, Kyushu University, Fukuoka, Japan

**Keywords:** Cell signalling, Molecular biology, Inflammation

## Abstract

Skeletal muscle mesenchymal progenitors (MPs) play a critical role in supporting muscle regeneration. However, under pathological conditions, they contribute to intramuscular adipose tissue accumulation, involved in muscle diseases, including muscular dystrophy and sarcopenia, age-related muscular atrophy. How MP fate is determined in these different contexts remains unelucidated. Here, we report that Ror2, a non-canonical Wnt signaling receptor, is selectively expressed in MPs and regulates their pathological features in a differential ligand-dependent manner. We identified Wnt11 and Wnt5b as ligands of Ror2. In vitro, Wnt11 inhibited MP senescence, which is required for normal muscle regeneration, and Wnt5b promoted MP proliferation. We further found that both Wnts are abundant in degenerating muscle and synergistically stimulate Ror2, leading to unwanted MP proliferation and eventually intramuscular adipose tissue accumulation. These findings provide evidence that Ror2-mediated signaling elicited by differential Wnts plays a critical role in determining the pathological fate of MPs.

## Introduction

Wnt signaling is critical in the regulation of organogenesis, tissue regeneration, and tumor development and progression. Binding of secreted Wnt ligands to Wnt receptors and co-receptors activates distinct signaling pathways: β-catenin-dependent canonical and β-catenin-independent non-canonical Wnt signaling. The Ror-family receptors, Ror1 and Ror2, can bind several Wnt ligands, including Wnt5a, Wnt5b, Wnt9a, Wnt9b, and Wnt11, to mediate non-canonical Wnt signaling attributable to similar or different biological responses, depending on the cellular context [1–6]. Thus, these non-canonical Wnt ligands and their receptors, Ror1 and Ror2, exhibit redundant or pleiotropic biological functions. However, the molecular mechanisms underlying their pleiotropic functions remain largely elusive. Although Ror1 and Ror2 are maintained at relatively low levels in adult tissues, accumulating evidence demonstrates that Ror1 and Ror2 expression increases in injured tissues to promote regeneration [7–9]. In addition, it has recently been shown that Ror1 and Ror2 are selectively expressed in adult stem cells, even in intact tissues, including hair follicles, dental pulp, and skeletal muscles [9–11].

Skeletal muscle has remarkable regenerative capacity. Following injury, satellite cells (SCs), which are skeletal muscle-specific tissue stem cells, start to proliferate and differentiate to generate new myofibers, thus playing an essential role in skeletal muscle regeneration. Mesenchymal progenitors (MPs), also known as fibro-adipogenic progenitors, are another type of tissue stem cells in skeletal muscle. Although MPs cannot differentiate into myofibers, in response to environmental cues, they secrete certain soluble factors to promote SC proliferation and differentiation, resulting in proper regeneration [12, 13]. It has recently been shown that MPs are important for skeletal muscle maintenance and hypertrophy [14–16]. However, they can also differentiate into adipocytes and fibroblasts, leading to intramuscular adipose tissue (IMAT) and fibrosis under pathological conditions in skeletal muscle [17, 18]. Since a plethora of MPs cause aberrant adipocyte and fibroblast accumulation, resulting in IMAT and fibrosis, coordinated regulation of MP proliferation, differentiation, and apoptosis is required for proper skeletal muscle regeneration [18, 19].

SC proliferation and differentiation are regulated by canonical and non-canonical Wnt signaling [20–24]. In this respect, it should be noted that Ror1 is expressed highly and selectively in SCs and plays a critical role in regulating SC proliferation during skeletal muscle regeneration [9]. Recent studies have indicated the possible role of non-canonical Wnt signaling activated by Wnt5a or Wnt7a in inhibiting adipogenic differentiation of MPs [25, 26]. Interestingly, it has been postulated that MPs are a significant source of Wnt ligands and can activate Wnt signaling via autocrine or paracrine machineries [25, 27]; however, little is known about the possible role of Ror-family receptors in regulating MPs. Therefore, we conducted an analysis using mouse skeletal muscles and their derived MPs to elucidate the role of Ror-family receptors in the functional regulation of MP. In this study, we report that Ror2 is selectively expressed in MPs. We explored the role of Ror2 in MPs through a combination of in vitro and in vivo analyses. For the first time, we provide evidence that distinct Wnt proteins synergistically activate Ror2 signaling and determine the pathological fate of MPs. Our findings reveal a novel diagnostic and therapeutic target for various muscular diseases whose therapeutic strategy remains unestablished.

## Results

### Ror2 plays a critical role in regulating MP proliferation, differentiation, and senescence

**Fig. 1:**
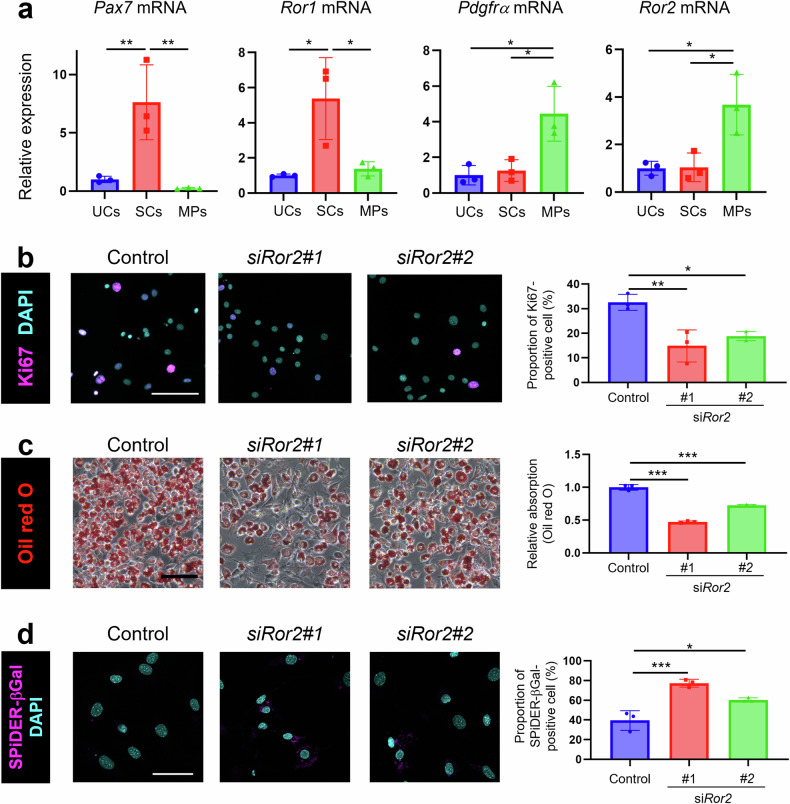
Ror2 is involved in regulating MP proliferation, differentiation, or cellular senescence. **a** Sorted satellite cells (SCs), mesenchymal progenitors (MPs), and unsorted cells (UCs) were obtained from (intact) skeletal muscles using FACS. Expression of *Pax7*, *Ror1*, *Pdgfrα*, and *Ror2* mRNAs in SCs, MPs, and UCs was measured using quantitative RT-PCR analysis (*n* = 3). **b** Representative images of MPs at 3 days after transfection with the respective siRNAs, followed by staining with anti-Ki-67 antibody (magenta) and DAPI (cyan). Scale bar: 100 μm. Right graph shows proportion of Ki-67-positive MPs (*n* = 3). **c** MPs, transfected with the respective siRNAs, followed by induction of adipogenic differentiation, were visualized using Oil Red O staining. Scale bar: 100 μm. Right graph shows relative absorption of Oil Red O at 6 days after induction of adipogenic differentiation (*n* = 3). **d** Representative images of MPs at 3 days after transfection with the respective siRNAs, followed by staining with SPiDER-βGal (magenta) and DAPI (cyan). Scale bar: 50 μm. Right graph shows proportion of SPiDER-βGal-positive cells (*n* = 3). Data in bar graphs are expressed as mean ± SD (*p < 0.05, **p < 0.01, ***p < 0.001, **a**: Holm’s test, **b**–**d**: Dunnett’s test).

We previously showed that Ror1 is highly expressed in SCs and contributes to skeletal muscle regeneration by promoting SC proliferation [9]. However, Ror2 function in skeletal muscle remained to be determined. Quantitative reverse transcription (qRT)-PCR analysis of SCs (SM/C-2.6-positive and CD31-, CD45-, Sca1-negative cells [28, 29]), MPs (Sca1-positive and CD31-, CD45-, SM/C-2.6-negative cells [12, 13]), and unsorted cells isolated from intact skeletal muscles using fluorescence-activated cell sorting (FACS) revealed that *Ror1* and *Pax7* (a representative SC marker) were selectively expressed in SCs, whereas *Ror2* and *Pdgfrα* (a representative MP marker) were predominantly expressed in MPs (Fig. 1a). To assess the proportion of Ror2-positive cells within the total MP population, we conducted fluorescence in situ hybridization analysis. When we defined MPs with three or more *Ror2* mRNA particles as Ror2-positive cells, the majority of sorted MPs (72.6%) were Ror2 positive (Supplementary Fig. 1a–c). These findings prompted us to explore the role of Ror2 in regulating MP function.

Isolated MPs were cultured in vitro and transfected with *Ror2* small interfering (si)RNAs to examine Ror2 function in MPs (Supplementary Fig. 2a). Anti-Ki-67 immunostaining and WST-8 assay revealed that Ror2 is required for MP proliferation (Fig. 1b and Supplementary Fig. 2b). Furthermore, *Ror2* knockdown (KD) inhibited the adipogenic differentiation of MPs, as assessed using Oil Red O staining and differentiation marker profiling (Fig. 1c and Supplementary Fig. 2c). Recent studies have shown that attenuated proliferation and adipogenic differentiation are representative phenotypes of MPs derived from aged mice [30, 31]. In this respect, it is of interest to note that *Ror2* KD induced MP senescence, as assessed using SPiDER-βGal and anti-p21 staining (Fig. 1d, Supplementary Fig. 2d, e). These findings indicated that Ror2 is required for MP proliferation, adipogenic differentiation, and inhibition of cellular senescence.

### Ror2 plays a crucial role in IMAT accumulation under muscle degeneration induced by glycerol but not cardiotoxin (Ctx) injection

**Fig. 2:**
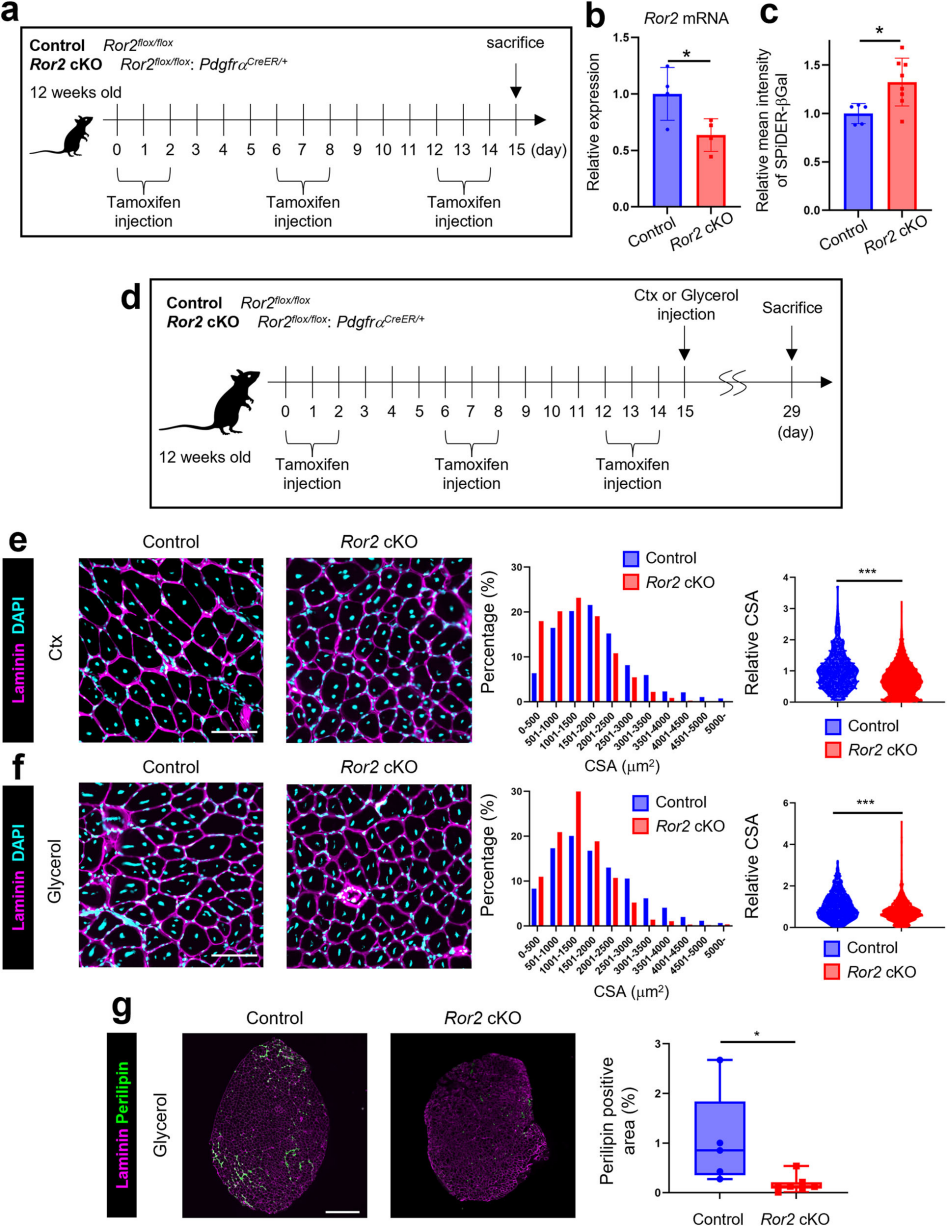
Ror2, expressed selectively in MPs, plays a crucial role in intramuscular adipose tissue (IMAT) accumulation. **a** Experimental design to analyze whether Ror2 inhibits cellular senescence of MPs in the skeletal muscles obtained from either control or *Ror2* cKO mice after intraperitoneal injection of tamoxifen. **b** Expression of *Ror2* in MPs isolated (sorted) from the skeletal muscles of either control or *Ror2* cKO mice was monitored using quantitative RT-PCR analysis (*n* = 3). **c** Fluorescence intensity of SPiDER-βGal in MPs sorted from the skeletal muscles of either control or *Ror2* cKO mice was monitored by flow cytometric analysis. Right graph shows relative mean fluorescence intensity of SPiDER-βGal in MPs isolated from the skeletal muscles of either control or *Ror2* cKO mice (control: *n* = 5, *Ror2* cKO: *n* = 8). **d** Experimental design to analyze the role of Ror2 in IMAT accumulation. Control and *Ror2* cKO mice were injected with either cardiotoxin (Ctx) or glycerol in the TA muscles after intermittent intraperitoneal injection of tamoxifen as indicated. Two weeks later, CSA of myofiber and accumulation of IMAT in the TA muscles were analyzed. Sections of the TA muscles, from either control or *Ror2* cKO mice 2 weeks after injection with Ctx (**e**) or glycerol (**f**), were stained with anti-laminin antibody (magenta) and DAPI (cyan). Scale bar: 100 μm. The middle histograms show the distribution of CSA in control and *Ror2* cKO mice. Right graphs show CSA of myofibers in the TA muscles from either control or *Ror2* cKO mice. (control: 960 myofibers from three animals (**e**) and 1061 myofibers from four animals (**f**), *Ror2* cKO: 2779 myofibers from five animals (**e**) and 1344 myofibers from four animals (**f**)). **g** TA muscles, from either control or *Ror2* cKO mice 2 weeks after injection with glycerol, were stained with anti-laminin (magenta) and anti-perilipin (green) antibodies. Scale bar: 600 μm. Right graph shows proportion of perilipin-positive area in the TA muscles. (control: *n* = 5, *Ror2* cKO: *n* = 7). (*p < 0.05, ***p < 0.001, **b**, **c**, **g**: Student’s *t*-test, **e**, **f**: Mann–Whitney test).

Next, we examined the role of Ror2 in MPs in vivo. As conventional *Ror2*-ablated mice die neonatally [32], we generated MP-specific *Ror2* cKO (*Ror2*^*flox/flox*^, *Pdgfrα*^*CreER/+*^) mice. To evaluate the KO efficiency, we separated MPs from intact muscles from control (*Ror2*^*flox/flox*^) and *Ror2* cKO mice after intermittent intraperitoneal injection of tamoxifen (Fig. 2a). *Ror2* expression was decreased in MPs from *Ror2* cKO mice compared with that in MPs from control mice (Fig. 2b). In addition, MP senescence was enhanced in *Ror2* cKO mice compared with that in control mice, as assessed using SPiDER-βGal staining, consistent with the data in vitro (Fig. 2c). However, IMAT levels and the relative proportions of SCs and MPs were comparable between control and *Ror2* cKO mice 6 months after tamoxifen injection (Supplementary Fig. 3a, b). To examine the role of Ror2 in IMAT accumulation in vivo, we injected the tibialis anterior (TA) muscles with Ctx or glycerol, which induces muscular injury without and with IMAT accumulation, respectively, after tamoxifen injection (Fig. 2d). The cross-sectional area (CSA) of myofibers from control mice was somewhat larger than that of myofibers from *Ror2* cKO mice after Ctx or glycerol injection (Fig. 2e, f). Importantly, while glycerol injection, not Ctx injection, induced IMAT accumulation in the TA muscles of control mice, IMAT accumulation induced by glycerol injection was significantly decreased in *Ror2* cKO mice compared with that in control mice (Fig. 2g). On the other hand, the degree of fibrosis induced by glycerol injection was not significantly affected (Supplementary Fig. 3c). These findings indicated that Ror2 in MPs plays a critical role in IMAT accumulation in the skeletal muscle in a context-dependent manner.

### Wnt5b-Ror2 signaling promotes MP proliferation in glycerol-injected skeletal muscle

**Fig. 3:**
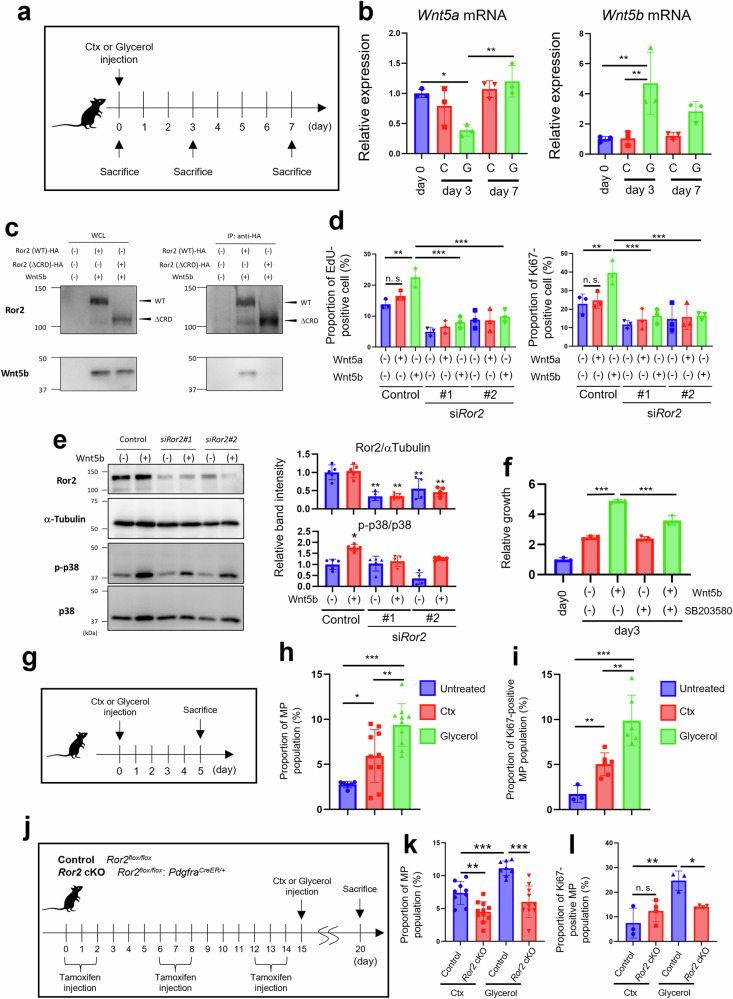
Wnt5b-Ror2 signaling promotes MP proliferation in glycerol-injected skeletal muscles. Expression of *Wnt5a* and *Wnt5b* in the TA muscles from untreated (day 0), Ctx (C)- or glycerol (G)-injected mice at the indicated time points (**a**) was analyzed using quantitative RT-PCR (**b**, *n* = 3). **c** Cos7 cells were transfected with the indicated plasmids. Subsequently, whole-cell lysates or anti-HA immunoprecipitates were prepared and subjected to western blotting (refer to Materials and Methods). **d** MPs, transfected with the indicated siRNAs, followed by stimulation with Wnt5a (200 ng/ml), Wnt5b (200 ng/ml), or their vehicle (0.1% BSA, designated as (-)) were stained with EdU or an anti-Ki-67 antibody. The proportion of EdU-positive (left) or Ki-67-positive (right) cells was quantified (*n* = 3). **e** Protein expression of Ror2, αTubulin, phosphorylated p38 (p-p38), and p38 proteins in MPs, transfected with the indicated siRNAs, and stimulated with either Wnt5b (200 ng/ml) or vehicle for 1 h. Right graphs show the relative band intensity of Ror2 and p-p38 normalized using αTubulin and p38, respectively (*n* = 5). **f** Proliferation of MPs stimulated with Wnt5b (200 ng/ml), in the presence or absence of a p38 inhibitor (SB203580 (10 mM)), was evaluated using WST-8 assay at the indicated time points (*n* = 3). Proliferation of MPs in TA muscles injected with either Ctx or glycerol was analyzed (**g**). Proportion of MPs (**h**) and Ki-67-positive MPs (**i**) in untreated, Ctx-, or glycerol-injected mice was monitored by flow cytometric analysis (untreated: *n* = 6 (**h**) and *n* = 3 (**i**), Ctx: *n* = 10 (**h**) and *n* = 6 (**i**), glycerol: *n* = 10 (**h**) and *n* = 6 (**i**)). Proliferation of MPs in TA muscles from control or *Ror2* cKO mice injected with either Ctx or glycerol after intermittent intraperitoneal injection of tamoxifen was analyzed (**j**). The proportion of MPs (**k**) and Ki-67-positive MPs (**l**) in TA muscles from control or *Ror2* cKO mice, injected with either Ctx or glycerol, was monitored by flow cytometric analysis (Ctx-injected control: *n* = 9 (**k**) and *n* = 3 (**h**), Ctx- injected *Ror2* cKO: *n* = 11 (**k**) and *n* = 4 (**l**), glycerol-injected control: *n* = 8 (**k**) and *n* = 3 (**l**), glycerol-injected *Ror2* cKO: *n* = 10 (**k**) and *n* = 4 (**l**)). Data in bar graphs are expressed as mean ± SD (*p < 0.05, **p < 0.01, ***p < 0.001, Holm’s test).

Ror1 and Ror2 selectively act as receptors for several Wnt ligands and thus mediate diverse cellular functions under physiological and pathological conditions. Although our findings indicated that Ror2 is a critical regulator of various cellular functions and MP fate, the responsible Wnt ligand(s) of Ror2 remained unclear. Therefore, we examined the expression profiles of the respective *Wnt* family genes in TA muscles treated with Ctx or glycerol to identify a candidate Wnt ligand(s) of Ror2 that may induce IMAT accumulation (Fig. 3a). Although Ror2 was required for IMAT accumulation (Fig. 2g), the expression of *Wnt5a*, a representative ligand of Ror2, was decreased on day 3 after glycerol injection (Fig. 3b, left panel). In contrast, the expression of *Wnt5b*, which encodes the closest relative of Wnt5a, was markedly increased in glycerol-, but not Ctx-, injected TA muscles (Fig. 3b, right panel). To investigate which type of cells express *Wnt5b* under pathological conditions, i.e., Ctx- or glycerol-injected TA muscles, we sorted MPs, SCs, a mixed population of endothelial cells and hematopoietic cells (CD31-, CD45-positive, and SM/C-2.6-, Sca1-negative), and the mixed others population (CD31-, CD45-, SM/C-2.6-, Sca1-negative) and subjected them to qRT-PCR analysis to evaluate *Wnt5b* expression. As shown, *Wnt5b* expression was primarily observed in MPs and SCs, and a significantly higher expression of *Wnt5b* was detected in MPs from glycerol-injected TA muscles than in those from Ctx-injected ones (Supplementary Fig. 4).

To examine Wnt5a and Wnt5b function in MPs, we treated MPs with *Wnt5a* siRNAs or *Wnt5b* siRNAs. *Wnt5a* KD did not affect MP proliferation, adipogenic differentiation, and senescence in our experimental settings (Supplementary Fig. 5a–e, see Materials and Methods). *Wnt5b* KD in MPs suppressed proliferation (Supplementary Fig. 5f–h) but not adipogenic differentiation and cellular senescence (Supplementary Fig. 5i, j). Consistent with a recent study [25], recombinant Wnt5a treatment inhibited the adipogenic differentiation of MPs (Supplementary Fig. 6). However, *Ror2* KD also inhibited adipogenic differentiation, suggesting that the other Wnt ligand(s) may promote adipogenic differentiation of MPs through Ror2 (Supplementary Fig. 6). Therefore, we examined how Wnt5b (presumably Wnt5b-Ror2 signaling) promotes MP proliferation. To this end, we examined whether Wnt5b can bind to Ror2 in Cos7 cells ectopically expressing Ror2 (wild-type) or Ror2 (ΔCRD) lacking the cysteine-rich domain (CRD), a putative Wnt protein-binding site, along with Wnt5b. We found that Wnt5b could associate with Ror2 via its extracellular CRD (Fig. 3c). Furthermore, anti-Ki-67 immunostaining and EdU incorporation analyses revealed that stimulation with recombinant Wnt5b, but not Wnt5a, promoted MP proliferation in a Ror2-dependent manner (Fig. 3d). Ror2 activates various signaling molecules, including MAPKs [33–35]; therefore, we analyzed the phosphorylation levels of the respective MAPKs in MPs following Wnt5b stimulation. While Jnk and Erk phosphorylation levels were unaffected by stimulation with Wnt5b (Supplementary Fig. 7), the p38 phosphorylation level increased in a Ror2-dependent manner (Fig. 3e). Consistent herewith, treatment with SB203580, a specific inhibitor of p38 [36], resulted in a significant suppression of Wnt5b-induced proliferation of MPs (Fig. 3f). We further examined whether glycerol injection can promote MP proliferation compared with Ctx injection in vivo (Fig. 3g). MP proliferation was significantly higher in glycerol-injected TA muscles than in Ctx-injected muscles (Fig. 3h, i). Furthermore, enhanced MP proliferation by glycerol injection into the TA muscles was markedly suppressed in *Ror2* cKO mice compared with that in control mice (Fig. 3j–l). Considering our finding that *Wnt5b* can be expressed in MPs and SCs under pathological conditions, these results indicated that Wnt5b-Ror2 signaling, activated by autocrine and/or paracrine machineries, promotes excessive MP proliferation in glycerol-injected muscle.

### Wnt11-Ror2 signaling plays a critical role in regulating MP proliferation, differentiation, and senescence

**Fig. 4:**
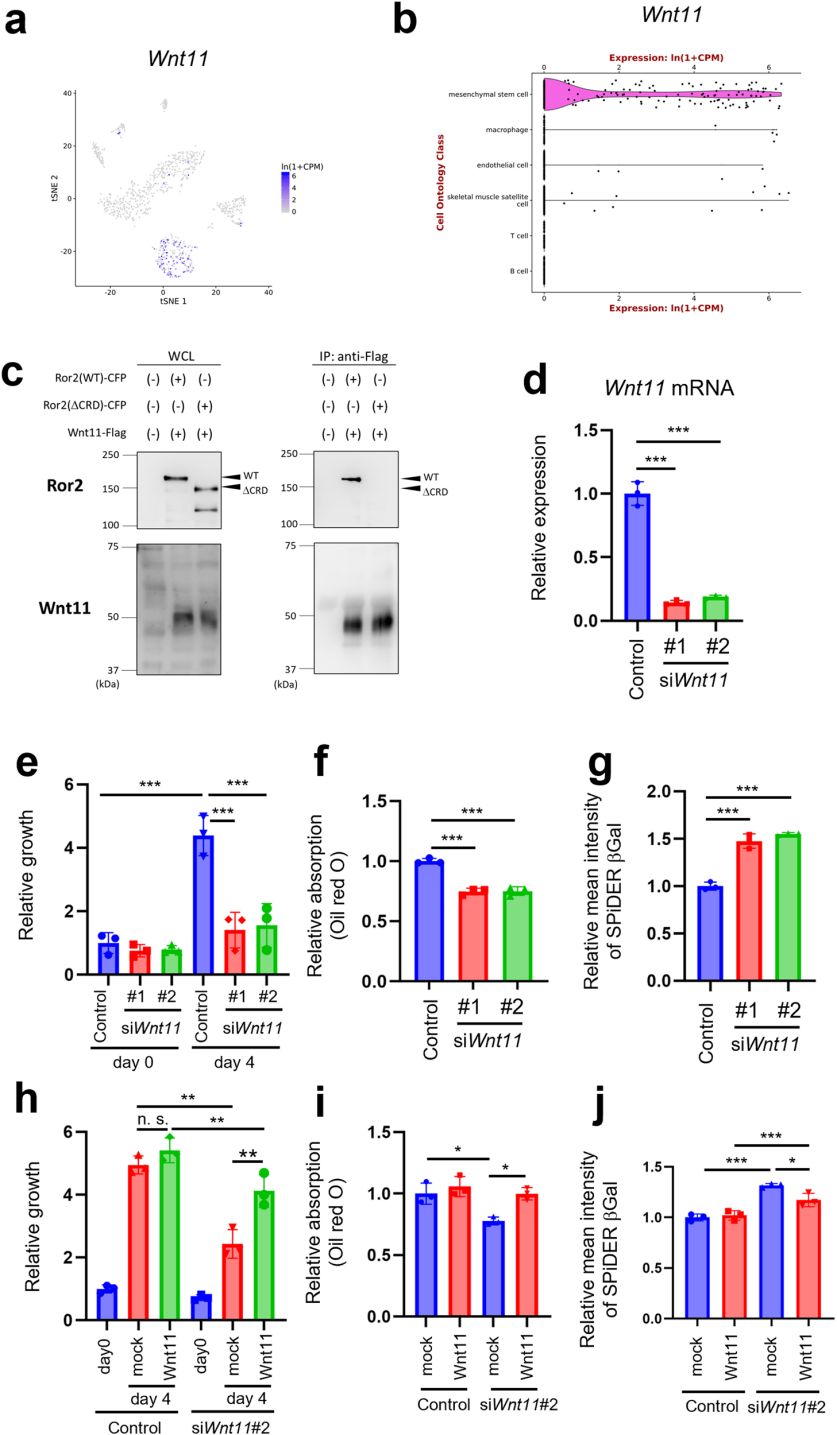
Wnt11 is involved in regulating MP proliferation, differentiation, or cellular senescence. **a** Expression of *Wnt11* was projected on t-SNE plots obtained from Tabula Muris. Colors span a gradient from blue (high expression) to gray (low expression). **b** Violin plot of *Wnt11* expressed in various cell ontology classes within limb muscles, as observed in the Tabula Muris dataset. **c** Cos7 cells were transfected with the indicated plasmids. Subsequently, whole-cell lysates or anti-Flag immunoprecipitates were prepared and subjected to western blotting. **d** Expression of *Wnt11* in isolated MPs treated with the indicated siRNAs was assessed using quantitative RT-PCR analysis (*n* = 3). **e** Proliferation of MPs, transfected with the indicated siRNAs, was quantified using the WST-8 assay at day 0 and day 4 post-transfection (*n* = 3). **f** MPs, transfected with the indicated siRNAs, were visualized using Oil Red O staining at 6 days after induction of adipogenic differentiation (*n* = 3). **g** Fluorescence intensity of SPiDER-βGal in MPs treated with the indicated siRNAs was monitored by flow cytometric analysis (*n* = 3). **h** MPs, which had been transfected with the specified siRNAs, were co-cultured indirectly with Cos7 cells that had been transfected with expression vectors encoding *mWnt11* or mock vectors. The proliferation of MPs was assessed using WST-8 assay at the indicated time points (*n* = 3). **i** MPs, which had been transfected with the indicated siRNAs, and then co-cultured indirectly with Cos7 cells transfected with expression vectors encoding *mWnt11* or mock, were visualized using Oil Red O at 6 days after induction of adipogenic differentiation (*n* = 3). **j** MPs, after transfection with the indicated siRNAs, were co-cultured indirectly with Cos7 cells that had been transfected with expression vectors encoding *mWnt11* or mock. Subsequently, the fluorescence intensities of SPiDER-βGal in MPs were monitored by flow cytometric analysis (*n* = 3). Data in bar graphs are expressed as mean ± SD (*p < 0.05, **p < 0.01, ***p < 0.001, n.s. not significant, **d**, **f**, **g**: Dunnett’s test, **e**, **h**–**j**: Holm’s test).

Although Ror2 was required for suppressing MP senescence (Figs. 1d, and 2c, Supplementary Fig. 2d, e), both Wnt5a and Wnt5b did not appear to be associated with cellular senescence (Supplementary Fig. 5e, j). Therefore, we surmised that another Wnt family protein(s) may act as a ligand(s) of Ror2 to inhibit MP senescence. It has recently been shown that acute injury induces MP senescence during the execution of proper tissue regeneration, whereas chronic injury inhibits MP senescence, resulting in detrimental tissue degeneration [37]. Indeed, we confirmed that MP senescence was mitigated in glycerol-injected muscles compared with that in Ctx-injected muscles (Supplementary Fig. 8). We surveyed Wnt proteins whose expression in TA muscle was inhibited by Ctx injection but sustained or enhanced by glycerol injection. Among the *Wnt* family genes examined, *Wnt11* expression decreased following Ctx injection but was sustained at a basal level in glycerol-injected TA muscles (Supplementary Fig. 9). Notably, the analysis of single-cell RNA sequencing data obtained from the Tabula Muris (https://tabula-muris.ds.czbiohub.org/) [38] database revealed that *Wnt11* was highly expressed in MPs (Fig. 4a, b). It was further confirmed that Wnt11 could associate with Ror2 via its extracellular CRD (Fig. 4c). Therefore, we hypothesized that Wnt11-Ror2 signaling, activated by autocrine or paracrine machinery, can inhibit senescence of MPs, while promoting their proliferation and adipogenic differentiation. We examined the possible role of Wnt11 in MP proliferation and adipogenic differentiation using WST-8 assay and Oil Red O staining. *Wnt11* KD inhibited MP proliferation and adipogenic differentiation (Fig. 4d–f) and enhanced their senescence (Fig. 4g). To examine whether Wnt11 stimulation could relieve the effects of *Wnt11* KD, we established Cos7 cells expressing Wnt11 (Cos7-Wnt11) and Cos7 cells transfected with a mock plasmid (Cos7-mock) as a control and performed indirect co-culture with MPs (transfected with *Wnt11* siRNAs or control siRNAs). Co-culture of *Wnt11*-KD MPs with Cos7-Wnt11 cells, but not Cos7-mock cells, resulted in (partial) restoration of suppressed proliferation and adipogenic differentiation and alleviation of enhanced cellular senescence (Fig. 4h–j). These results indicated that Wnt11-Ror2 signaling is required to regulate MP proliferation, adipogenic differentiation, and senescence.

### Wnt11-Ror2 signaling suppresses MP senescence by inhibiting nuclear membrane blebbing

**Fig. 5:**
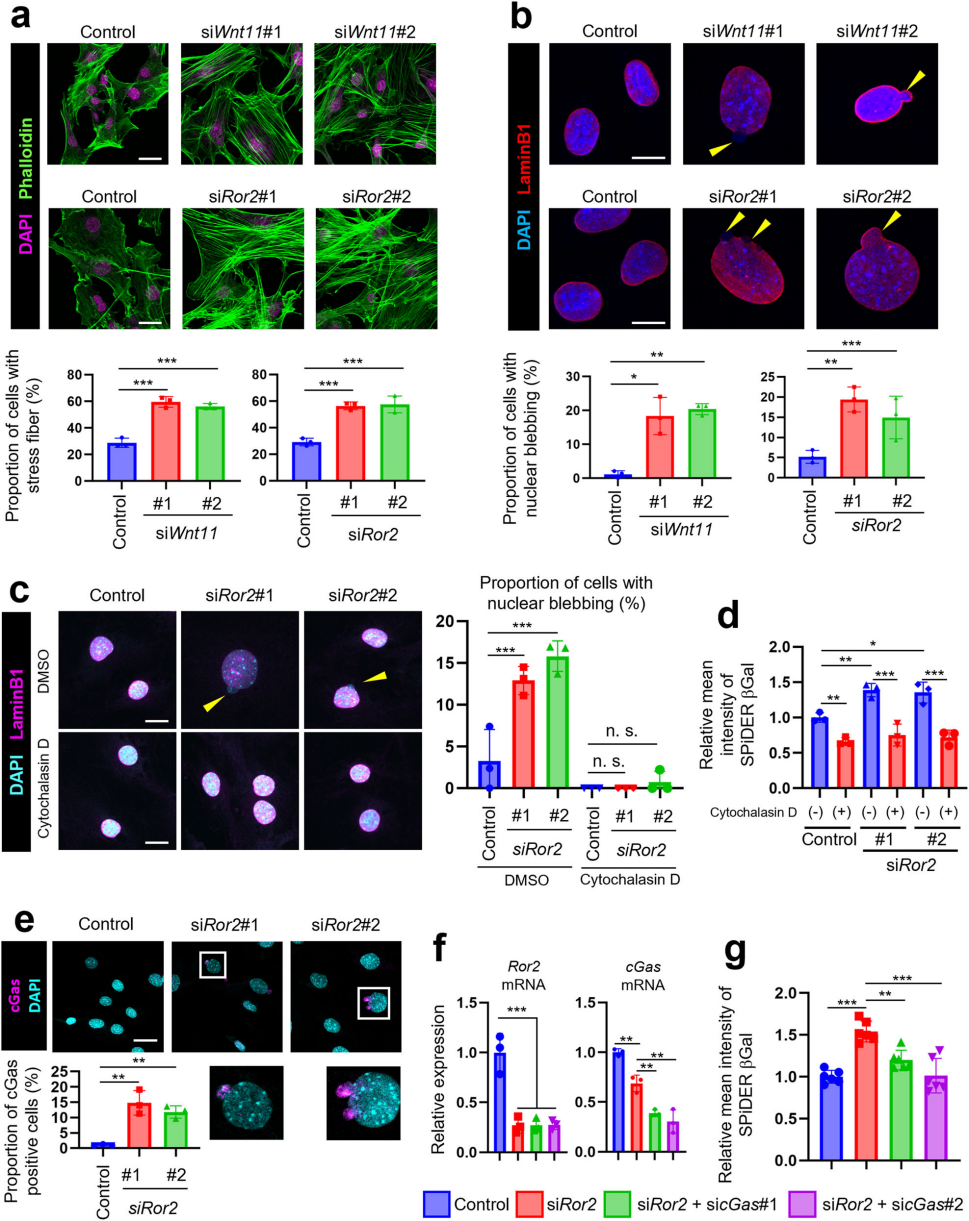
Wnt11-Ror2 signaling suppresses cellular senescence of MPs by maintaining nuclear membrane integrity. **a** Representative images of MPs, taken 2 days after transfection with the indicated siRNAs, were obtained following staining with Phalloidin (green) and DAPI (magenta). Scale bar: 25 μm. Lower graphs show proportion of cells with stress fiber (*n* = 3). **b** Representative images of MPs, taken at 3 days after transfection with the indicated siRNAs, were obtained following staining with an anti-lamin B1 antibody (red) and DAPI (blue). Scale bar: 10 μm. Lower graphs show proportion of cells with blebbed nuclear membrane (*n* = 3). **c** MPs, transfected with the indicated siRNAs, followed by treatment with cytochalasin D, were stained with an anti-lamin B1 antibody (magenta) and DAPI (cyan). Scale bar: 10 μm. Right graph shows proportion of cells with blebbed nuclear membrane (*n* = 3). **d** MPs, transfected with the indicated siRNAs, followed by treatment with cytochalasin D, were visualized using SPiDER-βGal. Fluorescence intensity of SPiDER-βGal in MPs was monitored by flow cytometric analysis. The graph shows relative mean fluorescence intensity of SPiDER-βGal (*n* = 3). **e** Representative images of MPs, at 3 days after transfection with respective siRNAs, were obtained following staining with an anti-cGas antibody (magenta) and DAPI (cyan). Scale bar: 25 μm. Lower graph shows proportion of cGas-positive cells (*n* = 3). **f** Expression of *Ror2* and *cGas* mRNAs in MPs treated with the indicated siRNAs was measured using quantitative RT-PCR analysis (*n* = 3). **g** Fluorescence intensity of SPiDER-βGal in MPs treated with the indicated siRNAs was monitored by flow cytometric analysis. The graph shows relative mean fluorescence intensity of SPiDER-βGal (*n* = 6). Data in bar graphs are expressed as mean ± SD (*p < 0.05, **p < 0.01, ***p < 0.001, **a**, **b**, **e**: Dunnett’s test, **c**, **d**, **f**, **g**: Holm’s test).

Next, we investigated the molecular mechanism underlying the induction of cellular senescence by *Wnt11* or *Ror2* KD. Wnt11-Ror2 signaling is involved in cell migration and actin remodeling [1, 39]. Therefore, we examined the effect of *Wnt11* or *Ror2* KD on actin cytoskeleton organization in MPs. KD of either *Wnt11* or *Ror2* resulted in drastic stress fiber formation throughout the cell, including the nuclear periphery (Fig. 5a). Accumulating evidence has demonstrated that perinuclear actin fibers induce nuclear blebbing [40, 41]; therefore, we examined morphological alterations of the nuclear membrane. Anti-lamin B1 immunostaining revealed that the proportion of MPs exhibiting nuclear blebbing was markedly increased upon *Wnt11* or *Ror2* KD (Fig. 5b). Furthermore, treatment of MPs with cytochalasin D, an inhibitor of actin polymerization, resulted in the inhibition of nuclear blebbing induced by *Ror2* KD (Fig. 5c). Intriguingly, cytochalasin D treatment also inhibited cellular senescence induced by *Ror2* KD (Fig. 5d). Self-genomic DNA extravasating into the cytosol activates the cGas-STING pathway [42, 43], which mediates cellular senescence induction [44]. In this respect, it should be noted that cGas was detected at the sites of nuclear blebs in *Ror2*-KD MPs (Fig. 5e) and that *cGas* KD mitigated MP senescence induced by *Ror2* KD (Fig. 5f, g). Based on these findings, we reasoned that Wnt11-Ror2 signaling suppresses cGas-mediated cellular senescence by inhibiting nuclear blebbing induced by perinuclear actin fibers.

### Wnt5b and Wnt11 synergistically promote MP proliferation and IMAT accumulation

**Fig. 6:**
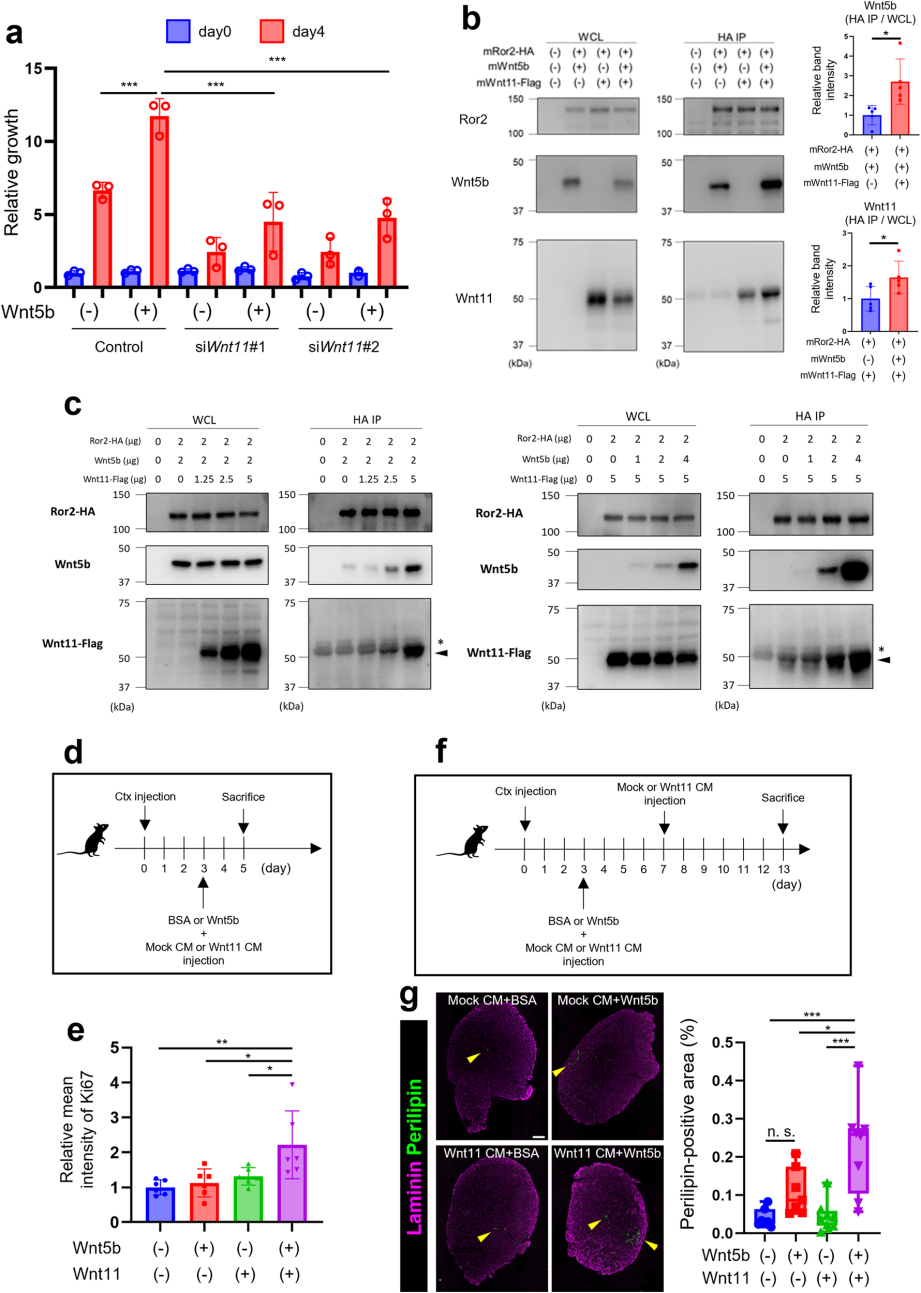
Co-stimulation with Wnt5b and Wnt11 induces IMAT accumulation in Ctx-injected skeletal muscles. **a** Proliferation of MPs, treated with respective siRNAs in the absence or presence of Wnt5b (200 ng/ml), was evaluated using WST-8 assay at the indicated time points (*n* = 3). **b** Cos7 cells were transfected with the indicated plasmids. Subsequently, whole-cell lysates (WCL) or anti-HA immunoprecipitates were prepared and subjected to western blotting. The graphs on the right show the relative band intensity of Wnt5b and Wnt11 in anti-HA immunoprecipitates, which were normalized by the corresponding levels of Wnt5b and Wnt11 in WCL (*n* = 5). **c** Cos7 cells were transfected with the respective plasmids at the indicated amounts. The amount of the plasmids transfected was adjusted to 9 μg (left) or 11 μg (right) in total with empty (mock) plasmid. Subsequently, WCL or anti-HA immunoprecipitates were prepared and subjected to western blotting. The representative data from one of two independent experiments are shown. The asterisks indicate nonspecific bands (Immunoglobulin heavy chain of antibodies), and triangles show the Wnt11-Flag bands. **d**, **e** Proportion of Ki-67-positive MPs in TA muscles treated with Wnt5b and/or Wnt11 CM after Ctx injection was monitored by flow cytometric analysis. The experimental design is shown in (**c**), and relative mean intensity of Ki-67 is shown in (**d**) (*n* = 6). **f**, **g** Experimental design to analyze the accumulation of IMAT in TA muscles treated with Wnt5b and/or Wnt11 CM after Ctx injection (**e**). Since the expression of Wnt5b was reduced on the 7th day after glycerol injection (see Fig. 4b) and the expression of Wnt11 was significantly suppressed on the 7th day following Ctx injection (see Fig. S5), Wnt11 CM, but not Wnt5b, was administered to the injured TA muscles not only on day 3 but also on day 7 after Ctx injection. The TA muscles treated with Wnt5b and/or Wnt11 CM after Ctx injection were stained with anti-laminin (magenta) and anti-perilipin (green) antibodies. Scale bar: 300 μm. The right graph in (**f**) shows proportion of perilipin-positive area in the TA muscles (BSA + mock CM: *n* = 7, Wnt5b + mock CM: *n* = 7, BSA + Wnt11 CM: *n* = 8, Wnt5b + Wnt11 CM: *n* = 8). Data in bar graphs are expressed as mean ± SD (*p < 0.05, **p < 0.01, ***p < 0.001, n.s. not significant, Holm’s test).

The finding that both *Wnt5b* and *Wnt11* were highly expressed in glycerol-injected muscles compared with those in Ctx-injected muscles raised the questions of how they regulate the versatility of Ror2-mediated signaling and whether they bind to Ror2 additively, competitively, or synergistically. We examined the involvement of Wnt11 in Wnt5b-induced proliferation of MPs. Wnt5b induced limited proliferation in *Wnt11*-KD MPs (Fig. 6a). Notably, when both Wnt5b and Wnt11 were present, their binding efficiency to Ror2 was increased (Fig. 6b). In fact, the binding of Wnt5b and Wnt11 to Ror2 was increased in the presence of increasing amounts of Wnt11 or Wnt5b (Fig. 6c), indicating that cooperative binding of Wnt5b and Wnt11 to Ror2 might be attributable to their synergistic action. On the other hand, Wnt5a failed to affect binding of Wnt11 to Ror2, and Wnt11 inhibited binding of Wnt5a to Ror2 (Supplementary Fig. 10). We next investigated whether simultaneous treatment with both Wnt5b and Wnt11 would enhance MP proliferation and IMAT accumulation in Ctx-injected muscle. The proportion of Ki-67-positive MPs increased upon simultaneous treatment with Wnt5b and Wnt11 in Ctx-injected muscle but was unaffected by treatment with Wnt5b or Wnt11 alone (Fig. 6d, e). Furthermore, treatment with both Wnt5b and Wnt11, but not with Wnt5b or Wnt11 alone, resulted in a pronounced IMAT accumulation in Ctx-injected muscles (Fig. 6f, g). These findings indicated that Wnt5b and Wnt11 synergistically contribute to excessive MP proliferation and IMAT accumulation.

## Discussion

SCs and MPs, which are tissue stem cells, play important roles in muscle regeneration, and dysfunction of these stem cells is thought to impair muscle homeostasis and cause muscle atrophy (sarcopenia) and degeneration due to aging or various muscular diseases. However, the functional regulation of these tissue stem cells remains largely unclear [20, 45]. We previously showed that Ror1, which functions as a receptor for the Wnt family of proteins, is selectively expressed in SCs, and inflammatory cytokines increase its expression upon skeletal muscle injury. Ror1 contributes to muscle regeneration by promoting SCs proliferation [9]; however, the role of Ror-family receptors in MP regulation remains elusive. In this study, we found that Ror2 expressed in MPs is critical for IMAT accumulation. By comparing a glycerol-induced skeletal muscle injury model (with IMAT accumulation) and a Ctx-induced skeletal muscle injury model (with limited IMAT formation), we found that Wnt5b and Wnt11 act as Ror2 ligands, rather than the well-known Ror2 ligand, Wnt5a. Wnt11-Ror2 signaling promotes MP proliferation and adipogenic differentiation and inhibits cellular senescence by regulating stress fiber-induced nuclear blebbing, associated with cGas-STING pathway activation. In contrast, Wnt5b-Ror2 signaling activates p38 MAPK, thereby promoting MP proliferation. Wnt11 and Wnt5b bind to the extracellular CRD of Ror2 expressed on MPs to activate Wnt11-Ror2 and Wnt5b-Ror2 signaling, respectively. Interestingly, both Wnts bind to the Ror2 CRD via synergistic, and not competitive, machinery. We further found that MP proliferation and IMAT accumulation are promoted when both signaling pathways are activated simultaneously in MPs.

We assume that the cellular response and fate of MPs are determined by the condition where Wnt11 alone, Wnt5b alone, or both Wnt proteins bind to Ror2 on MPs. As the spatiotemporal dynamics of Wnt11 and Wnt5b production and distribution are predicted to differ depending on qualitative differences in skeletal muscle injury and the etiology/pathology of various muscular diseases, it will be important to investigate their spatiotemporal production and distribution patterns in muscular diseases. In this study, we found that Wnt5b and Wnt11 are expressed highly in glycerol-injected muscle compared to Ctx-injected one. It has been shown that glycerol injection elicits anti-inflammatory macrophage activation and induces production of anti-inflammatory cytokines compared to Ctx injection [46]. Consistently, pro-inflammatory cytokines, such as TNFα, IL-1α, and IL-1β, can inhibit IMAT accumulation [47]. On the other hand, IL-4-polarized anti-inflammatory macrophages promote adipogenic differentiation of MPs [48]. These findings indicate that the inflammatory response in Ctx- or glycerol-injected muscle might differ. Therefore, it is important to investigate whether the difference in inflammation might affect specific Wnt expression patterns.

Although Wnt5b and Wnt11 could bind cooperatively to Ror2, Wnt5a failed to affect the binding of Wnt11 to Ror2. Interestingly, the presence of Wnt11 inhibited the binding of Wnt5a to Ror2. At present, it remains unclear about the molecular mechanisms underlying the binding of Wnt5a, Wnt5b, and Wnt11 to Ror2 as well as Ror2-mediated signaling elicited by these Wnt proteins. Furthermore, it should be noted that Ror2 and Frizzled-family receptors, another type of Wnt receptors, associate via their extracellular CRDs [49]. Thus, Ror2 may activate different signaling pathways by associating with different Frizzled receptors in MPs. Further study will be required to confirm this.

Although a previous study using conventional Ror1-, Ror2-, and their double KO has shown that Ror1 and Ror2 exhibit functional redundancy and pleiotropy [47], there is no report on their functional redundancy and pleiotropy in adult tissue stem cells. Our study demonstrated the selective/independent expression and functions of Ror1 and Ror2 in two different tissue stem cell types in adult skeletal muscles, SCs and MPs. Notably, while it appeared that Wnt5a, a representative Ror1 ligand, was not implicated in Ror1-mediated signaling in SCs [9], the findings of the present study on MPs draw attention to the potential involvement of Wnt family protein(s) other than Wnt5a in Ror1-mediated signaling in SCs.

Although glycerol-induced skeletal muscle injury is a commonly used model for studying muscle degeneration, it may not completely encompass the spectrum of muscular diseases. Therefore, future investigations should aim to determine whether Ror2 signaling, activated by Wnt5b and Wnt11, exacerbates pathological conditions, utilizing alternative pathological models such as *mdx* mice. In addition, we assessed the importance of Wnt5b and Wnt11 in IMAT accumulation through the administration of these Wnts in a Ctx-induced skeletal muscle injury model. We further revealed that expression of *Wnt5b*, but not that of *Ror2*, *Wnt5a*, and *Wnt11*, is significantly higher in the skeletal muscles from patients with Duchenne muscular dystrophy (DMD) than in those from unaffected control using deposited dataset in Gene Expression Omnibus (GSE38417) (https://www.ncbi.nlm.nih.gov/geo/) (Supplementary Fig. 11). It will be of interest to examine how much extents Ror2 signaling, activated synergistically by Wnt5b and Wnt11, will contribute to the progression of muscular diseases accompanied with IMAT accumulation including DMD and sarcopenia.

It is well established that intercellular crosstalk between SCs and MPs plays a critical role in regulating skeletal muscle maintenance and regeneration [13, 14, 50]. It is expected that the molecular mechanisms underlying this crosstalk will be elucidated by focusing on Ror1 in SCs, Ror2 in MPs, and secreted proteins, including various Wnt proteins (e.g., Wnt5b and Wnt11). Finally, our findings indicate that Wnt5b-Ror2 signaling in MPs can serve as a suitable diagnostic and therapeutic target for sarcopenia and various muscular diseases, including DMD.

## Materials and methods

### Animal experiments

Male C57BL/6N mice (8–12 weeks of age, 20–25 g body weight) were purchased from Japan SLC (Shizuoka, Japan). Breeding colonies were kept at 22–25 °C and humidity of 40–65%. All mice were maintained under a 12-/12-h light/dark cycle. The maximum housing density was five mice. All materials, including cage, feeders, bottles, and water, were autoclaved. To induce muscle injury and IMAT accumulation, Ctx (Latoxan, Valence, France) or glycerol (Nacalai Tesque, Kyoto, Japan) was injected into skeletal muscle [9, 13]. Briefly, Ctx (2.5 μl of 10 μM Ctx/g body weight) or glycerol (50 μl of 50% (v/v) glycerol/TA muscle) was injected into the TA muscles. Two weeks after injection, the TA muscles were harvested, and immunofluorescence staining was performed to analyze the CSA of myofibers and the proportion of the perilipin-positive area. *Ror2*^*flox/flox*^ mice were generated as described previously [51]. To investigate the roles of Wnt5b and Wnt11 in IMAT accumulation after muscle injury, recombinant Wnt5b (4 μg/ml, R&D Systems, Minneapolis, MN) and/or conditioned medium of Cos7 cells stably expressing Wnt11 (see details below) were/was injected into the TA muscles after Ctx injection. *Pdgfra*^*CreER/+*^ mice were obtained from Jackson Laboratory (stock #018280, Bar Harbor, ME). *Ror2*^*flox/flox*^; *Pdgfra*^*CreER/+*^ mice (10–14 weeks of age) were intraperitoneally injected with tamoxifen (100 μg/g body weight) (Sigma-Aldrich, St. Louis, MO) once a day for 9 days to suppress *Ror2* expression in MPs. In total, 1978 mice were used for this study (including mice used to generate transgenic and gene-edited mice, to explore optimal conditions for experiments, and to learn experimental techniques). All animal experiments were approved by the Institutional Animal Care and Use Committee (permission numbers: P171011-R1 and P220904) and conducted at the Institute for Experimental Animals, Kobe University Graduate School of Medicine, according to the Kobe University Animal Experimentation Regulations.

### Isolation of SCs and MPs

Bilateral TA, soleus, gastrocnemius, quadriceps femoris, and gluteus maximus muscles were digested in Dulbecco’s Modified Eagle Medium (DMEM)/F-12 (FUJIFILM Wako Pure Chemical Corporation, Osaka, Japan) containing 0.5% (w/v) collagenase type II (Worthington, Lakewood, NJ) at 37 °C for 90 min and then filtered through a 40-μm nylon mesh (pluriSelect Life Science, Leipzig, Germany). The cell suspensions were washed with ice-cold phosphate-buffered saline (PBS) containing 2% (v/v) fetal bovine serum (FBS). SCs and MPs were isolated from the cell suspension by FACS (FACS Aria III; BD Biosciences, Franklin Lakes, NJ) using anti-CD31, CD45, Sca-1, and SM/C-2.6 antibodies (antibody list is provided in Supplementary Table 1). SCs can be identified as SM/C-2.6-positive and CD31-, CD45-, and Sca-1-negative cells. MPs can be identified as Sca-1-positive and CD31-, CD45-, and SM/C-2.6-negative cells.

### MP culture and siRNA transfection

MPs were isolated from bilateral TA, soleus, and gastrocnemius muscles 3 days after Ctx injection using FACS as described above and cultured in DMEM (FUJIFILM Wako Pure Chemical Corporation) supplemented with 10% (v/v) FBS, 50 U/ml penicillin (Meiji Seika Pharma, Tokyo, Japan), 50 μg/ml kanamycin (FUJIFILM Wako Pure Chemical Corporation), and 10 ng/ml basic fibroblast growth factor (FUJIFILM Wako Pure Chemical Corporation) at 37 °C in the presence of 5% CO_2_ and 3% O_2_ for 3–4 days. The MPs were used for in vitro experiments at 37 °C in the presence of 5% CO_2_ and 20% O_2_.

MPs were transfected with siRNAs using Lipofectamine RNAiMAX reagent (Thermo Fisher Scientific, Waltham, MA) following the manufacturer’s instructions. Briefly, siRNAs (siRNAs produced by Sigma-Aldrich: 20 nM, siRNAs produced by Thermo Fisher Scientific: 10 nM) were mixed with RNAiMAX reagent in OPTI-MEM (Thermo Fisher Scientific), incubated at 20–25 °C for 10 min, and added to cultured MPs. The sequences of the siRNAs are listed in Supplementary Table 2.

### Proliferation assay

To examine the proportion of Ki-67-positive MPs, MPs were transfected with the indicated siRNAs (siRNA sequences are listed in Supplementary Table 2) and cultured in DMEM containing 10% (v/v) FBS for 3 days. The cells were subsequently fixed with 4% (w/v) paraformaldehyde (PFA) at 20–24 °C for 10 min. Fixed MPs were stained with anti-Ki-67 antibody and 4′,6-diamidino-2-phenylindole to evaluate the percentage of Ki-67-positive MPs.

To investigate the proportion of EdU-positive MPs, MPs were transfected with the indicated siRNAs and cultured in DMEM containing 10% (v/v) FBS for 3 days. Subsequently, the MPs were treated with EdU (10 μM, Thermo Fisher Scientific) for 30 min and then fixed with 4% (w/v) PFA. EdU incorporation was detected using a Click-it EdU Cell Proliferation Kit for Imaging (Thermo Fisher Scientific) according to the manufacturer’s instructions.

For WST-8 assay, MPs were seeded in triplicate in 96-well plates (1000 cells/well), transfected with the indicated siRNAs, and cultured for the indicated periods. WST-8 reagent (Cell Counting Kit-8; Dojindo Laboratories, Kumamoto, Japan) was added to the medium, and the plates were incubated for 90 min, after which the absorbance was measured at 450 nm.

To assess the involvement of p38 in Wnt5b-induced proliferation, a p38 inhibitor (10 μM, SB203580, Cayman Chemical, Ann Arbor, MI) and recombinant Wnt5b (200 ng/ml, R&D Systems) were added 1 day after seeding MPs in 96-well plates. Subsequently, WST-8 assay was performed as described above.

### Adipogenic differentiation of MPs

MPs were cultured in adipogenic induction medium (DMEM containing 10% [v/v] FBS, 0.5 mM IBMX [Sigma-Aldrich], 0.25 μM dexamethasone [Sigma-Aldrich], and 10 μg/ml insulin [Sigma-Aldrich]) for 3 days, followed by culture in adipogenic maintenance medium (DMEM containing 10% FBS [v/v] and 10 μg/ml insulin) for 3 days.

### Oil Red O staining

To stain adipocytes, cells seeded in a 24-well plate (50,000 cells/well) were fixed with 4% (w/v) PFA at 20–24 °C for 10 min. The fixed cells were rinsed with 60% (v/v) isopropanol and stained with Oil Red O (FUJIFILM Wako Pure Chemical Corporation) in 60% (v/v) isopropanol at 20–24 °C for 30 min. The stained cells were rinsed with 60% (v/v) isopropanol and then incubated in 100% isopropanol for 20 min to extract the Oil Red O incorporated into adipocytes. Subsequently, the absorbance of Oil Red O in the supernatant at 490 nm was measured.

### SPiDER-βGal analysis

To detect senescent cells, MPs transfected with the indicated siRNAs were cultured in DMEM supplemented with 10% (v/v) FBS for 3 days and then stained with SPiDER-βGal (Dojindo Laboratories) according to the manufacturer’s instructions. Briefly, 1 μM SPiDER-βGal working solution was added to an MP culture, which was then incubated at 37 °C for 30 min. The fluorescence intensity or proportion of SPiDER-βGal-positive cells was determined using flow cytometry or microscopy.

### Gene overexpression

Cos7 cells (obtained from ATCC (Manassas, VA)) were cultured in DMEM supplemented with 10% (v/v) FBS at 37 °C in the presence of 5% CO_2_ and 20% O_2_. An expression plasmid (pcDNA3 or pECFP) encoding mRor2-HA or mRor2-CFP (wild-type or DCRD) and expression plasmids (pCMV5) encoding mWnt5b and/or mWnt11-Flag were co-transfected into Cos7 cells using ViaFect (Promega, Madison, WI).

### Establishment of Cos7 cells stably expressing mWnt11

Cos7 cells were co-transfected with an expression plasmid (pCMV5) encoding mWnt11 and a plasmid encoding puromycin resistance (pBabe-Puro) at a 10:1 ratio. Transfected cells were selected using puromycin and assessed for *mWnt11* expression to obtain cells expressing mWnt11 (Cos7-Wnt11). Cos7 cells transfected with empty vector (pCMV5) and pBabe-Puro were also selected using puromycin (Cos7-mock).

### Preparation of conditioned medium

To prepare conditioned medium, 5 × 10^5^ Cos7-Wnt11 or Cos7-mock cells were cultured in 10-cm-diameter dishes in FBS-free DMEM for 2 days. The medium was harvested; centrifuged at 4 °C, 500 × *g* for 5 min to remove dead cells; and concentrated using Vivaspin 20-10 K (Cytiva, Marlborough, MA). The concentrated conditioned medium was injected into injured TA muscles.

### Indirect co-culture

For indirect co-culture, 0.4-μm-pore polyester membrane Transwell inserts in 12- or 24-well plates (Costar, Cambridge, MA, USA) were used to allow MPs to share the medium with Cos7-Wnt11 or Cos7-mock cells without any direct cell–cell contact.

### Flow cytometry

Cells were collected, resuspended in PBS containing 2% (v/v) FBS, and incubated with the respective antibodies on ice for 1 h. Flow cytometric analysis was performed using a BD LSRFortessaTM X-20 instrument (BD Biosciences). The data were analyzed using the FlowJo software (version 10. 7. 1, BD Biosciences).

### Immunofluorescence analysis

Isolated TA muscles were frozen with isopentane, cooled with liquid nitrogen, and sectioned (20 mm) in a cryostat (CM1860; Leica, Wetzlar, Germany). The frozen sections were permeabilized with 0.2% (v/v) Triton X-100 in PBS, blocked with 3% (w/v) BSA in PBS, and incubated with primary antibodies at 4 °C overnight. The sections were washed with PBS and incubated with secondary antibodies at 20–24 °C for 1 h, mounted with Fluoro-KEEPER (Nacalai Tesque), and observed under a fluorescence microscope (BZ-X700; Keyence, Osaka, Japan). TA muscle CSA and the proportion of the perilipin-positive area were analyzed using ImageJ (version v1.54 g, National Institutes of Health, Bethesda, MD).

Cultured cells were fixed with 4% (w/v) PFA for 10 min and permeabilized with 0.2% (v/v) Triton X-100. After washing with PBS, the cells were blocked with 3% (w/v) BSA in PBS for 1 h and incubated with primary antibodies at 4 °C overnight. The cells were washed with PBS and incubated with secondary antibodies at 20–24 °C for 1 h. The stained cells were mounted with Fluoro-KEEPER and observed using the LSM700 microscope (Carl Zeiss, Oberkochen, Germany). The antibodies used are listed in Supplementary Table 1.

### Fluorescence in situ hybridization

Fluorescence in situ hybridization was conducted to investigate Ror1 expression in MPs. MPs, isolated from intact skeletal muscle by FACS, were spun down onto a glass slide by centrifugation at 20–24 °C using Plate Spin II (Kubota, Tokyo, Japan). Subsequently, MPs were fixed with 4% (w/w) PFA for 10 min at 20–24 °C and then subjected to fluorescence in situ hybridization. The fluorescence in situ hybridization was conducted using the QuantGene ViewRNA in situ hybridization cell kit (Affymetric, Santa Clara) with an *Ror2* mRNA probe designed by Affymetrics for hybridization to mouse *Ror2* mRNA, in accordance with the manufacturer’s instructions.

### Western blotting

Cells were solubilized with lysis buffer (50 mM Tris-HCl [pH 7.5], 150 mM NaCl, 1% [v/v] Nonidet P-40, 1 mM EDTA, 10 mM NaF, 1 mM Na_3_VO_4_, 10 μg/ml aprotinin [Takara Bio, Shiga, Japan], 10 μg/ml leupeptin [PEPTIDE INSTITUTE, INC., Osaka, Japan], and 1 mM *p*-APMSF [FUJIFILM Wako Pure Chemical Corporation]). Proteins (10 μg/lane) were separated using sodium dodecyl sulfate (SDS)-polyacrylamide gel electrophoresis (PAGE) and transferred onto Immobilon-P membranes (Merck Millipore, Darmstadt, Germany). The membranes were blocked with 3% (v/w) BSA in TBST (TBS [137 mM NaCl, 2.7 mM KCl, 25 mM Tris] containing 0.05% [v/v] Tween-20) for 1 h, followed by immunoblotting with primary antibodies (Supplementary Table 1) at 4 °C overnight. Then, the membranes were washed with TBST at 20–24 °C for 30 min, followed by immunoblotting with secondary antibodies (Supplementary Table 1) at 20–24 °C for 1 h. Finally, the membranes were washed with TBST at 20–24 °C for 30 min, and immunoreactive bands were visualized using ImmunoStar LD (FUJIFILM Wako Pure Chemical Corporation) and a LAS 1000 imaging system (GE Healthcare, Chicago, IL). Relative band intensity was determined using the ImageJ software.

### Immunoprecipitation

Cells were solubilized with lysis buffer (50 mM Tris-HCl [pH 7.5], 150 mM NaCl, 0.5% [v/v] Nonidet P-40, 5 mM EDTA, 50 mM NaF, 1 mM Na_3_VO_4_, 10 μg/ml aprotinin [Takara Bio], 10 μg/ml leupeptin [PEPTIDE INSTITUTE, INC.], and 1 mM *p*-APMSF [FUJIFILM Wako Pure Chemical Corporation]). The cell lysates were immunoprecipitated with anti-green fluorescent protein or anti-Flag antibody and Dynabeads Protein G (Thermo Fisher Scientific). The immunoprecipitates were washed with lysis buffer and eluted with sample buffer (62.5 mM Tris-HCl [pH 6.8], 5% [v/v] 2-mercaptoethanol, 2% [w/v] SDS, 5% [w/v] sucrose, and 0.005% [w/v] bromophenol blue). Proteins from immunoprecipitates and whole-cell lysates were separated using SDS-PAGE and transferred onto Immobilon-P membranes. The membranes were immunoblotted with the respective antibodies (Supplementary Table 1) and immunoreactive bands visualized as described above.

### qRT-PCR

Total RNA was extracted from cultured cells using Sepasol-RNA I SuperG (Nacalai Tesque), and its quality was assessed using a NanoDrop spectrophotometer (Thermo Fisher Scientific). Subsequently, the extracted RNA was reverse transcribed into cDNA using the PrimeScript RT Reagent (Takara Bio). qPCRs were run in a LightCycler 480 II instrument (Roche, Basel, Switzerland). Expression levels of genes of interest were normalized to those of the 18S ribosomal RNA gene. The sequences of the primers used are listed in Supplementary Table 3.

### Analysis using public datasets

Expression patterns of *Pax7*, *Pdgfrα*, *Ror1*, *Ror2*, and *Wnt11* in various cell types comprising skeletal muscle were investigated using Tabula Muris [38], a single-cell RNA sequencing database (https://tabula-muris.ds.czbiohub.org/). T-SNE and violin plots were drawn using the tool on the website. Data of *Wnt5b* expression in skeletal muscle biopsy from patients with DMD and unaffected control was obtained from Gene Expression Omnibus (GSE38417) (https://www.ncbi.nlm.nih.gov/geo/).

### Statistical analysis

Data were analyzed using GraphPad Prism 9.0 (GraphPad Software, Boston, MA). Data are presented as mean ± standard deviation. Means of two groups were compared using the unpaired two-tailed Student’s *t*-test, and means of three or more groups were compared using one-way ANOVA followed by Holm’s or Dunnett’s test. In cases where data exhibited a non-normal distribution, the Mann–Whitney test was performed. Significance was set to *p* values < 0.05. No statistical methods were used to predetermine the sample size. Each sample size is indicated in the figure or legend. Any samples or animals were not excluded from the analysis. Randomization and blinding were not used.

## Supplementary information

Supplementary information

Original western blot

## Data Availability

The data in Supplementary Fig. 9 can be acquired from Gene Expression Omnibus (GSE38417) (https://www.ncbi.nlm.nih.gov/geo/). The data of single-cell RNA-sequence was obtained from Tabula Muris (https://tabula-muris.ds.czbiohub.org/) database.
