# Supplementary material for: Ror2 signaling regulated by differential Wnt proteins determines pathological fate of muscle mesenchymal progenitors
Source: Cell Death Dis. 2024 Oct 29;15(10):784. doi: 10.1038/s41419-024-07173-9 (PMC11519583; doi:10.1038/s41419-024-07173-9)
Supplement: Supplementary file 1 — Supplementary information [file 41419_2024_7173_MOESM1_ESM.docx]

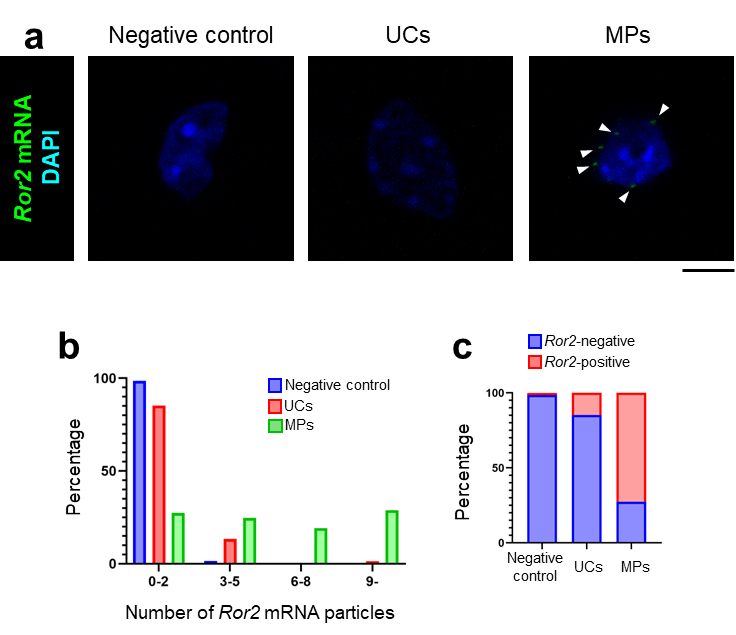
**Supplementary Information**

**Supplementary Figure 1. Analysis of single-cell RNA** **sequencing from Tabula Muris**

**a.** Representative images of fluorescence *in situ* hybridization analysis. White arrowheads indicate *Ror2* mRNA particles (green). Negative control represents MPs treated without the primary probe. Scale bar: 5 μm. **b.** Histogram showing the distribution of the number of intracellular *Ror2* mRNA particles. **c.** Cells with three or more *Ror2* mRNA particles were classified as *Ror2*-positive. The proportions of *Ror2*-positive and *Ror2*-negative cells are presented. Negative control: n = 67, UCs: n = 75, MPs: n = 73.


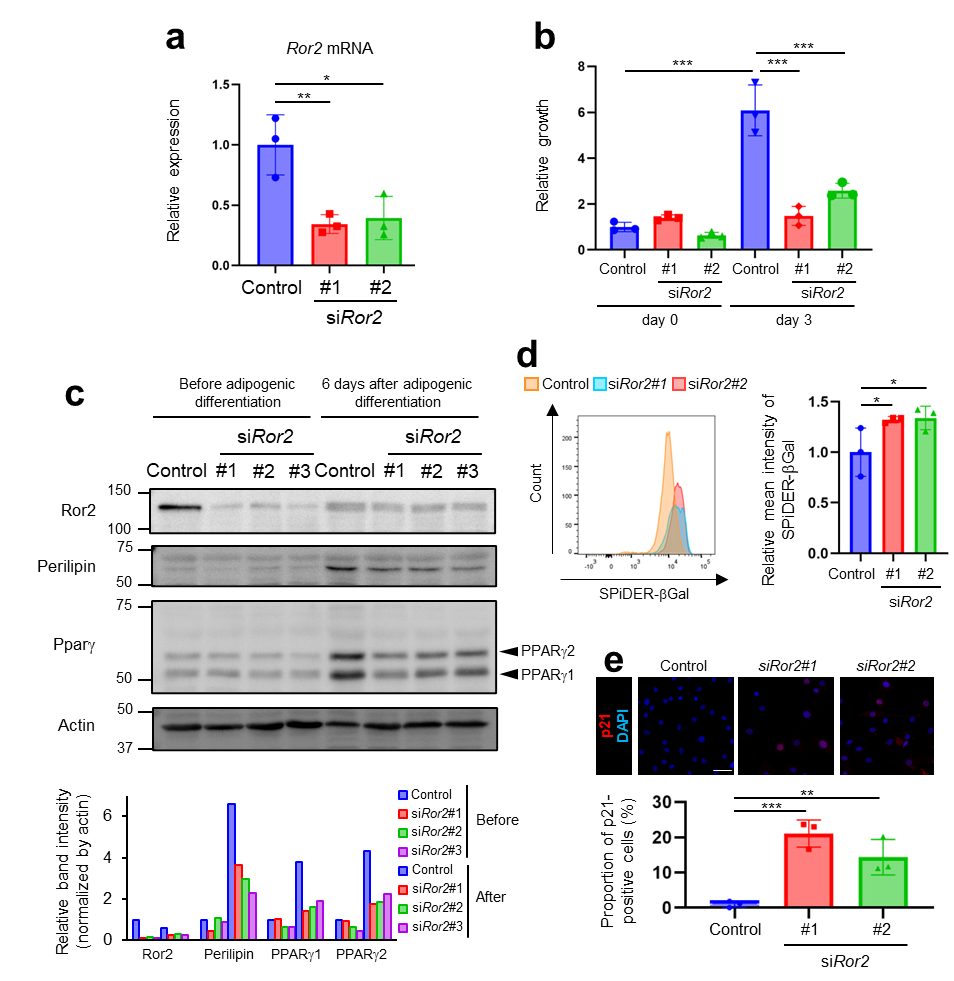


**Supplementary Figure 2. Ror2 plays critical roles in regulating various functions of MPs.**

**a.** Expression of *Ror2* in isolated MPs treated with the indicated siRNAs was analyzed using quantitative RT-PCR (n = 3). **b.** Proliferation of MPs transfected with the indicated siRNAs was assessed using the WST-8 assay (n = 3). **c.** Expression of Ror2, Perilipin, Pparγ, and Actin proteins in MPs, transfected with the indicated siRNAs, followed by induction of adipogenic differentiation, was analyzed using western blotting. Lower graph shows relative band intensity of Ror2, Perilipin, and Pparγ proteins, normalized by the intensity of Actin. Representative results are shown here (n = 2). **d.** Fluorescence intensity of SPiDER-βGal in MPs treated with indicated siRNAs was monitored by flow cytometric analysis. The right graph shows the relative mean fluorescence intensity of SPiDER-βGal (n = 3). **e.** Representative images of MPs at 3 days after siRNA transfection, visualized using staining with an anti-p21 antibody (red) and DAPI (blue). Scale bar: 50 μm. Lower graph shows proportion of p21-positive cells (n = 3). Data in bar graphs are expressed as mean ± S. D. (**p* < 0.05, ***p* < 0.01, ****p* < 0.001, a, d, e: Dunnett’s test, b: Holm’s test).


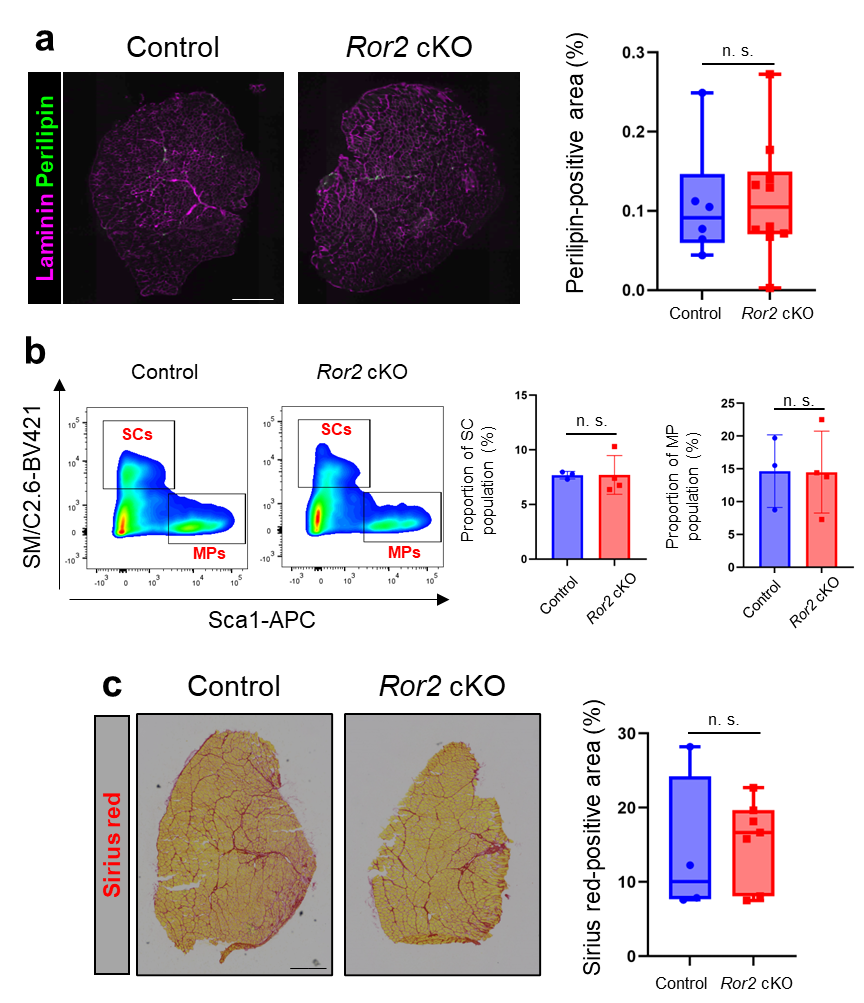


**Supplementary Figure 3. Phenotypic analyses of *Ror2* cKO mice.**

**a.** TA muscles, from control or *Ror2* cKO mice bred for 6 months after intraperitoneal injection of tamoxifen, were stained with anti-Laminin (magenta) and anti-perilipin (green) antibodies. Scale bar: 600 μm. The right graph shows the proportion of perilipin-positive area in the TA muscles (control: n = 6, *Ror2* cKO: n = 10). **b.** Proportion of SCs and MPs in the skeletal muscles from control or *Ror2* cKO mice bred for 6 months after intraperitoneal injection of tamoxifen was examined by flow cytometric analysis. The left panel shows representative results of flow cytometric analysis. The right graphs show proportion of SCs and MPs (control: n = 3, *Ror2* cKO: n = 4). **c**. TA muscles, from either control or *Ror2* cKO mice 2 weeks after injection with glycerol, were stained with Sirius red. Scale bar: 600 μm. Right graph shows Sirius red positive area in the TA muscles. (control: n = 4, *Ror2* cKO: n = 7). Data in bar graphs are expressed as mean ± S. D. (n. s.: not significant, Student’s *t*-test).

**
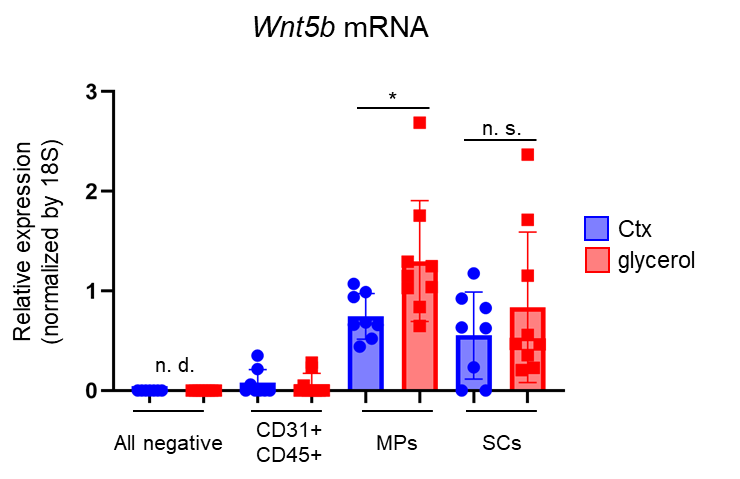
**

**Supplementary Figure 4. Analysis of *Wnt5b* expression in the cells sorted from the injured skeletal muscles.**

Expression of *Wnt5b* in MPs, SCs, a mixed population of endothelial cells and hematopoietic cells (CD31-, CD45-positive, and SM/C-2.6-, Sca1-negative), and the mixed others population (CD31-, CD45-, SM/C-2.6-, Sca1-negative) from Ctx- or glycerol-injected TA muscles was analyzed by quantitative RT-PCR (All negative (Ctx): n = 7, All negative (glycerol): n = 8, CD31+/CD45+ (Ctx): n = 8, CD31+/CD45+ (glycerol): n = 9, MPs (Ctx): n = 8, MPs (glycerol): n = 9, SCs (Ctx): n = 8, SCs (glycerol): n = 9). Data in the bar graph are expressed as mean ± S. D. (n. d.: not detected, **p* < 0.05, n. s.: not significant, Holm’s test).


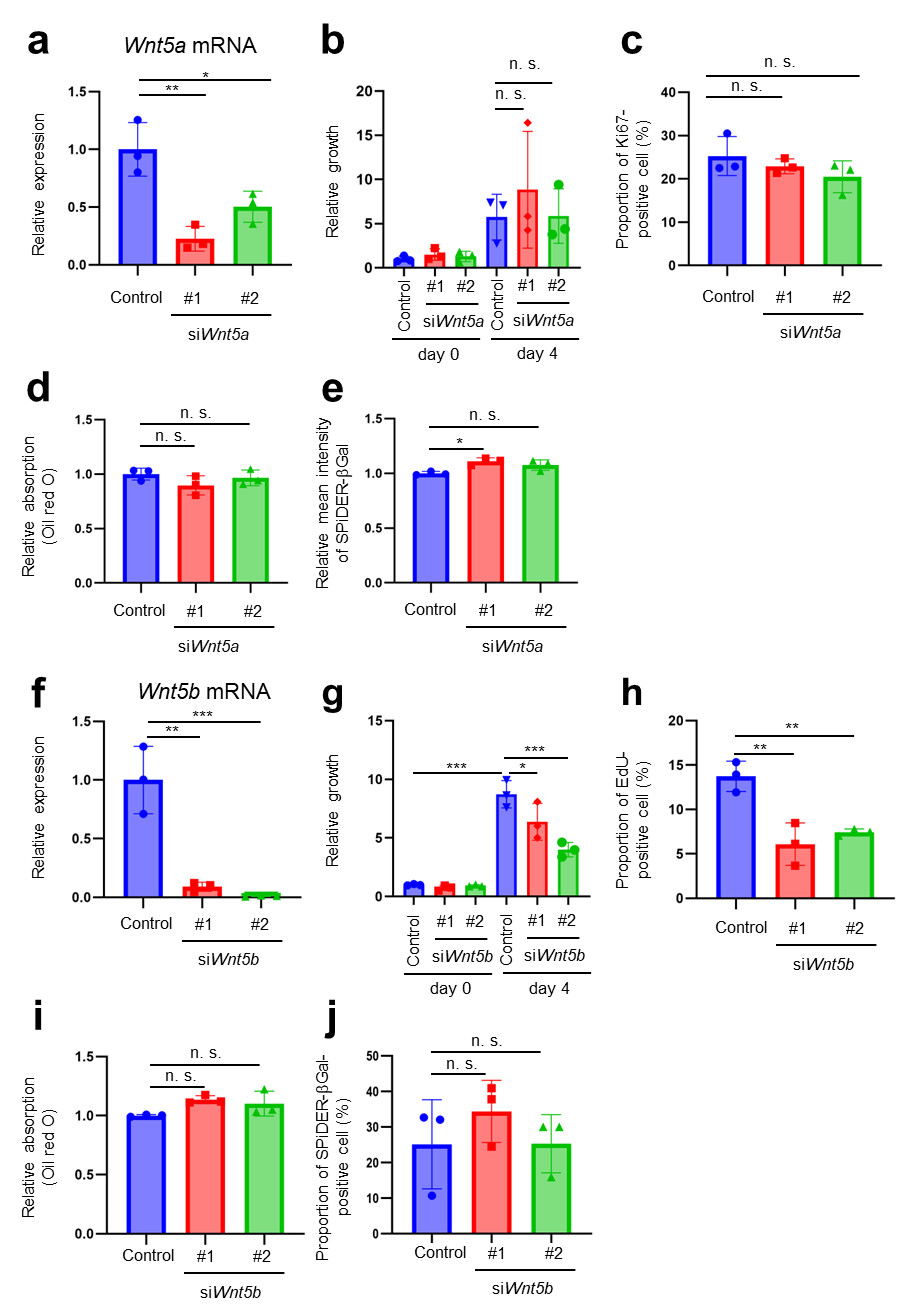


**Supplementary Figure 5. Role of intrinsic Wnt5a and Wnt5b in regulating MP proliferation, adipogenic differentiation, and cellular senescence.**

**a.** Expression of *Wnt5a* in MPs treated with the indicated siRNAs was analyzed using quantitative RT-PCR (n = 3). **b.** Proliferation of MPs transfected with the indicated siRNAs was measured using the WST-8 assay at the indicated time points (n = 3). **c.** Proportion of Ki-67-positive MPs transfected with the indicated siRNAs was analyzed (n = 3). **d.** MPs, transfected with the indicated siRNAs, were stained using Oil Red O at 6 days after induction of adipogenic differentiation (n = 3). **e.** Fluorescence intensity of SPiDER-βGal in MPs treated with indicated siRNAs was monitored by flow cytometric analysis. The graph shows relative mean fluorescence intensity of SPiDER-βGal (n = 3). Data in bar graphs are expressed as mean ± S. D. (**p* < 0.05, ***p* < 0.01, n. s.: not significant, a, c, d, e: Dunnett’s test, b: Holm’s test). **f.** Expression of *Wnt5b* in MPs treated with the indicated siRNAs was analyzed using quantitative RT-PCR (n = 3). **g.** Proliferation of MPs transfected with the indicated siRNAs was evaluated using WST-8 assay at the indicated time points (n = 3). **h.** Proportion of EdU-positive MPs transfected with the indicated siRNAs was analyzed (n = 3). **i.** MPs, transfected with the indicated siRNAs, were stained using Oil Red O at 6 days after induction of adipogenic differentiation (n=3). **j.** Proportion of SPiDER-βGal-positive MPs at 3 days after transfection with the indicated siRNAs was assessed by flow cytometric analysis. The graph shows relative mean fluorescence intensity of SPiDER βGal (n = 3). Data in bar graphs are expressed as mean ± S. D. (**p* < 0.05, ***p* < 0.01, ****p* < 0.001, n. s.: not significant, a, c, d, e: Dunnett’s test, b: Holm’s test)


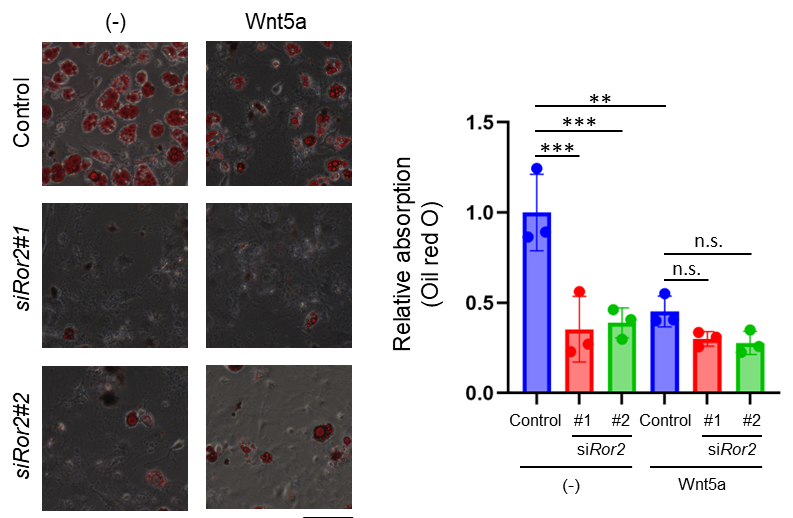


**Supplementary Figure 6. Treatment with recombinant Wnt5a inhibits adipogenic differentiation of MPs.**

MPs, after being transfected with the respective siRNAs, were exposed to recombinant Wnt5a (at a final concentration of 200 ng/ml) diluted with 0.1% BSA or treated with the vehicle alone (0.1% BSA, indicated as (-)) as a control. After 3 days, adipogenic differentiation was initiated, and the cells were subsequently visualized using Oil Red O staining. Scale bar: 100 μm. The right graph shows the relative absorption of Oil Red O at 6 days after induction of adipogenic differentiation (n = 3). Data in bar graphs are expressed as mean ± S. D. (***p* < 0.01, ****p* < 0.001, Holm’s test).


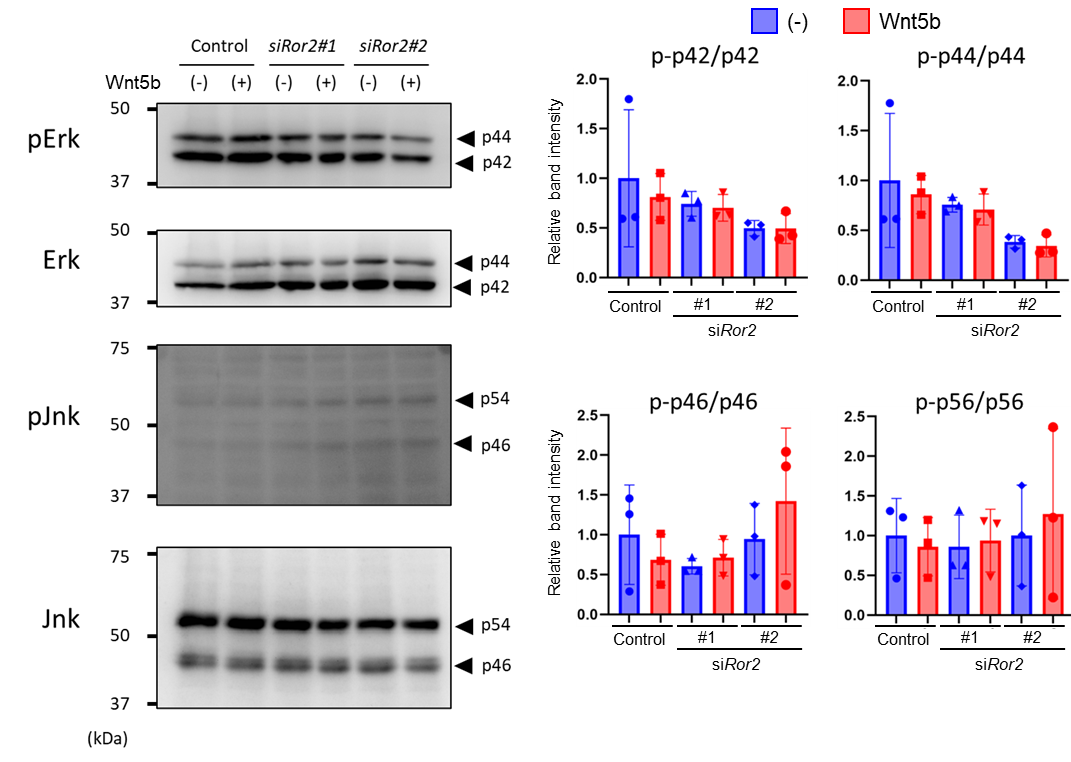


**Supplementary Figure 7. Wnt5b-Ror2 signaling fails to promote phosphorylation of Erk and Jnk in MPs.**

Protein expression of phosphorylated Erk (pErk, Thr202/Tyr204), Erk, phosphorylated Jnk (pJnk, Thr183/Tyr185), and Jnk proteins in MPs, transfected with the indicated siRNAs, and stimulated with either Wnt5b (200 ng/ml) or vehicle for 1 h. Right graphs show the relative band intensity of p-p42, p-p44, p-p46, and p-p54 normalized using p42, p44, p46, and p54, respectively (n = 3).


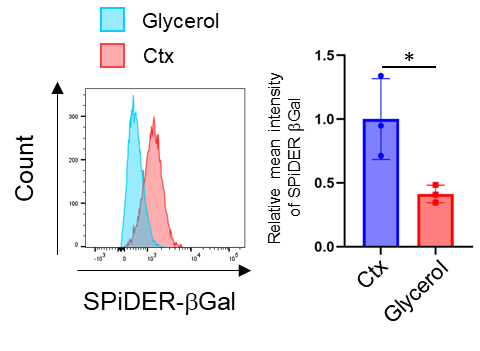


**Supplementary Figure 8. Glycerol injection inhibits cellular senescence of MPs.**

Fluorescence intensity of SPiDER-βGal in MPs sorted from either Ctx- or glycerol-injected TA muscles was monitored by flow cytometric analysis (left). Right graph shows relative mean fluorescence intensity of SPiDER-βGal in MPs isolated from either Ctx- or glycerol-injected TA muscles (n = 3). Data in bar graphs are expressed as mean ± S. D. (**p* < 0.05, Student’s *t*-test)


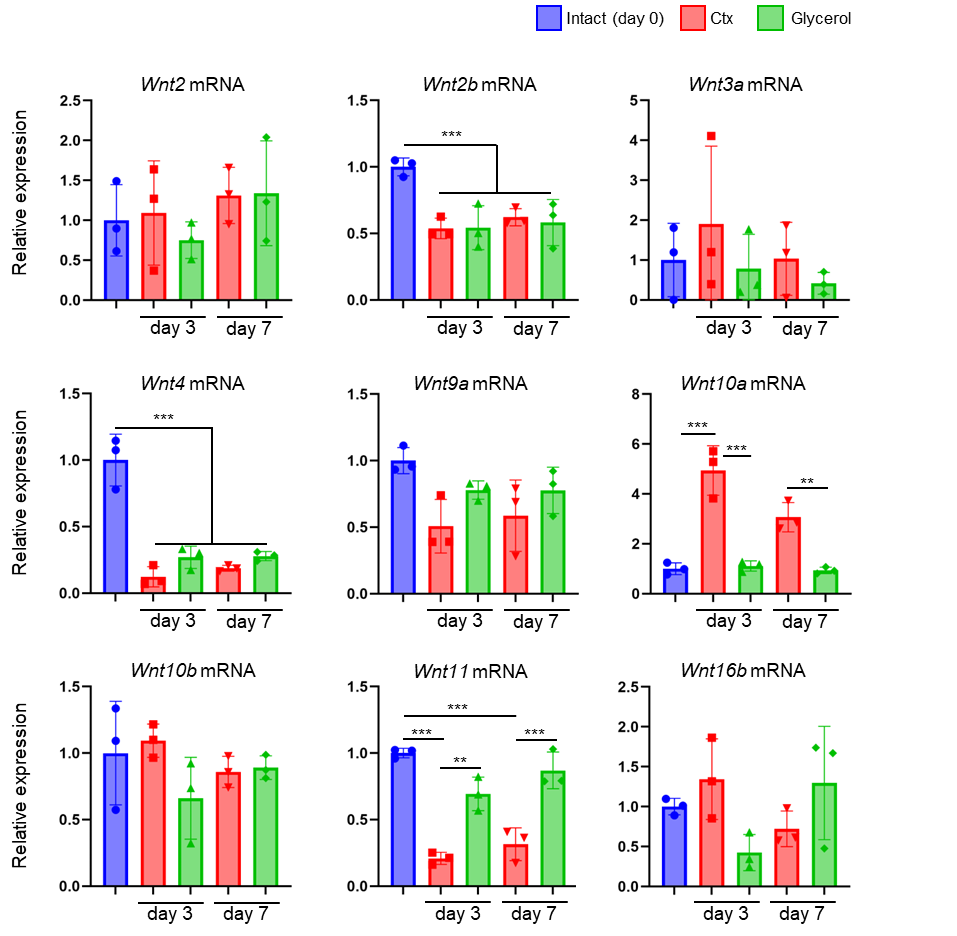


**Supplementary Figure 9. Comparative expression of the respective *Wnt* transcripts in TA muscles injected with either Ctx or glycerol.**

Expression of *Wnt2, Wnt2b, Wnt3a, Wnt4, Wnt9a, Wnt10a, Wnt10b*, *Wnt11*, and *Wnt16b* in TA muscles obtained from untreated (day 0), Ctx-injected, or glycerol-injected mice at the indicated time points was analyzed using quantitative RT-PCR (n = 3). Data in bar graphs are expressed as mean ± S. D. (**p* < 0.05, ***p* < 0.01, ****p* < 0.001, Holm’s test)


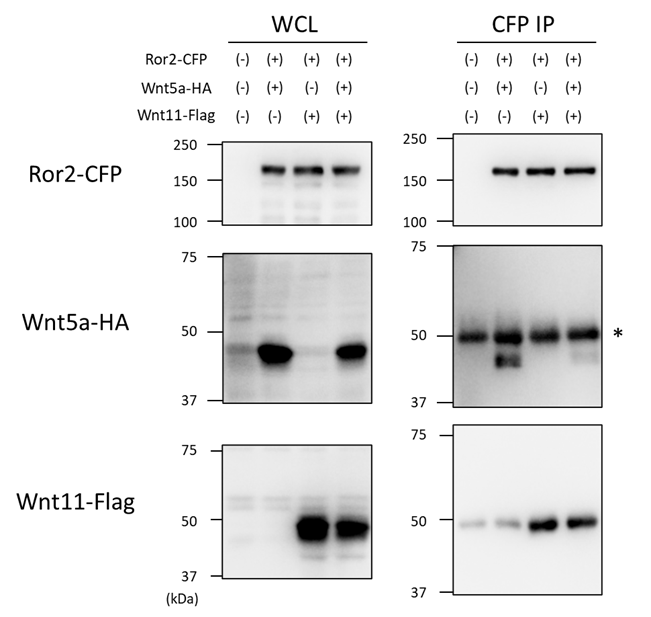


**Supplementary Figure 10. Wnt5a and Wnt11 do not exhibit cooperative binding to Ror2.**

Cos7 cells were transfected with the indicated plasmids. Subsequently, whole-cell lysates (WCL) or anti-CFP immunoprecipitates were prepared and subjected to western blotting. The representative data from one of two independent experiments are shown. The asterisks indicate nonspecific bands.


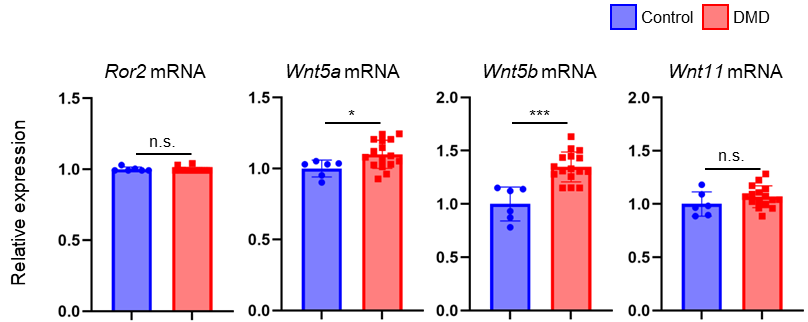


**Supplementary Figure 11. *Wnt5b* expression in patients with Duchenne muscular dystrophy (DMD) is higher than that in unaffected control.**

Expression of *Ror2*, *Wnt5a*, *Wnt5b*, and *Wnt11*  in skeletal muscle biopsy from patients with DMD and unaffected control was examined using GSE38417 dataset in Gene Expression Omnibus database (https://www.ncbi.nlm.nih.gov/geo/). Data in bar graphs are expressed as mean ± S. D. (Control: n = 6, DMD: n = 16) (*p<0.05, ****p* < 0.001, Student’s *t*-test)

**Supplementary Table 1. List of antibodies**

| **Antigen** | **Source** | **Catalog no.** | **Dilution** |
| --- | --- | --- | --- |
| Ki-67 | Thermo Fisher | 14-5698-82 | IF: 1/200 |
| Ki-67-BV421 | BioLegend | 652411 | FCM: 1/100 |
| Laminin | R&D System | MAB4656 | IF: 1/200 |
| Perilipin | Cell Signaling | 9349 | IF: 1/200  WB: 1/500 |
| CFP | Clontech | 632381 | IP: 1/1000  WB: 1/1000 |
| Ror2 | Our laboratory |  | WB: 1/500 |
| Wnt5b | Abcam | Ab93134 | WB: 1/500 |
| Flag | Merck | F1804 | IP: 1/1000 |
| Flag | Proteintech | 20543-1-AP | WB: 1/500 |
| HA | Abcam | ab187915 | IP: 1/1000 |
| αTubulin | MBL | PM054 | WB: 1/500 |
| p38 | Cell Signaling | 8690 | WB: 1/500 |
| Phosphorylated p38 | Cell Signaling | 4511S | WB: 1/500 |
| Erk | Cell Signaling | 4696S | WB: 1/500 |
| Phosphorylated Erk | Cell Signaling | 9101S | WB: 1/500 |
| Jnk | Cell Signaling | 9252S | WB: 1/500 |
| Phosphorylated Jnk | Cell Signaling | 9251S | WB: 1/500 |
| cGas | Cell Signaling | 31659 | IF: 1/200 |
| Lamin B1 | Abcam | ab16048 | IF: 1/200 |
| Pparg | Cell Signaling | 2443 | WB: 1/500 |
| p21 | Santa Cruz | sc-6246 | IF: 1/200 |
| Actin | Santa Cruz | sc-8432 | WB: 1/500 |
| CD31-FITC | BioLegend | 102406 | FCM: 1/200 |
| CD31-PE | BioLegend | 102407 | FCM: 1/100 |
| CD45-FITC | Thermo Fisher | 11-0451-85 | FCM: 1/200 |
| CD45-PE | BioLegend | 103106 | FCM: 1/100 |
| Sca1-APC | BioLegend | 108112 | FCM: 1/100 |
| SM/C-2.6 | Dr. So-ichiro Fukada |  | FCM: 1/400 |
| **Secondary antibodies** | | | |
| Anti-rabbit HRP | Bio-Rad | 1706515 | WB: 1/2000 |
| Anti-mouse HRP | Bio-Rad | 1706516 | WB: 1/2000 |
| Anti-mouse Alexa 488 | Thermo Fisher | A11029 | IF: 1/500 |
| Anti-mouse Alexa 546 | Thermo Fisher | A11030 | IF: 1/500 |
| Anti-rabbit Alexa 488 | Thermo Fisher | A11034 | IF: 1/500 |
| Anti-rabbit Alexa 546 | Thermo Fisher | A11035 | IF: 1/500 |
| Anti-rat Alexa 647 | Thermo Fisher | A21247 | IF: 1/500 |

IF: Immunofluorescence, FCM: Flow cytometry, IP: Immunoprecipitation, WB: Western blotting

**Supplementary Table 2. Sequence of siRNAs**

| **Gene** | **Sequence (5′-3′)** | **siRNA ID** | **Source** |
| --- | --- | --- | --- |
| *Ror2* #1 | (sense) CCCUUGAGCAUGAUCUUCA  (anti-sense) UGAAGAUCAUGCUCAAGGG | s77263 | Thermo Fisher |
| *Ror2* #2 | (sense) CAAUUUUCAGGAUGACGAU  (anti-sense) AUCGUCAUCCUGAAAAUUG | s77264 | Thermo Fisher |
| *Ror2* #3 | (sense) GCAGAUUACUACAAACUCA  (anti-sense) UGAGUUUGUAGUAAUCUGC | s77265 | Thermo Fisher |
| *Wnt5a* #1 | (sense) GUCAGAAGUAUAUAUCAUA  (anti-sense) UAUGAUAUAUACUUCUGAC | s76087 | Thermo Fisher |
| *Wnt5a* #2 | (sense) GGUGGUCUCUAGGUAUGAA  (anti-sense) UUCAUACCUAGAGACCACC | s76088 | Thermo Fisher |
| *Wnt5b* #1 | (sense) CCGUGUAUAAGAUGGCUGA  (anti-sense) UCAGCCAUCUUAUACACGG | 00111083 | Sigma-Aldrich |
| *Wnt5b* #2 | (sense) GUGCCAACACCAGUUUCGA  (anti-sense) UCGAAACUGGUGUUGGCAC | 00111084 | Sigma-Aldrich |
| *Wnt11* #1 | (sense) GAUGCUCCUAUGAAGGUGA  (anti-sense) UCACCUUCAUAGGAGCAUC | 00179252 | Sigma-Aldrich |
| *Wnt11* #2 | (sense) CUGAUGCGUCUACACAACA  (anti-sense) UGUUGUGUAGACGCAUCAG | 00179254 | Sigma-Aldrich |
| *cGas* #1 | (sense) GCUACUAUGAACAUGUGAA  (anti-sense) UUCACAUGUUCAUAGUAGC | 00129826 | Sigma-Aldrich |
| *cGas* #2 | (sense) CAAUCUAUUCUCUCAAGAA  (anti-sense) UUCUUGAGAGAAUAGAUUG | 00129827 | Sigma-Aldrich |

**Supplementary Table 3. List of qRT-PCR primers**

| **Gene** | **Forward primer (5′-3′)** | **Reverse primer (5′-3′)** |
| --- | --- | --- |
| *Ror1* | GCTGCGGATTAGAAACCTTG | TACGGCTGACAGAATCCATC |
| *Ror2* | ATGTGGACTCCCTCCAGATG | GAAGACGAAGTGGCAGAAGG |
| *Pax7* | CTGGATGAGGGCTCAGATGT | GGTTAGCTCCTGCCTGCTTA |
| *Pdgfra* | TGAATCCTGCAGACGAGAGC | ATGTCCATGTAGTCGCCGTT |
| *Wnt2* | ATCTCTTCAGCTGGCGTTGT | CCTTCCTTCCAGCTCTGTTG |
| *Wnt2b* | CGTTCGTCTATGCTATCTCGTCAG | ACACCGTAATGGATGTTGTCACTAC |
| *Wnt3a* | CCCTTTCCAGTCCTGGTGTA | CTTGAAGAAGGGGTGCAGAG |
| *Wnt4* | CTGGAGAAGTGTGGCTGTGA | CAGCCTCGTTGTTGTGAAGA |
| *Wnt5a* | CAAATAGGCAGCCGAGAGAC | CTCTAGCGTCCACGAACTCC |
| *Wnt5b* | ATGCCCGAGAGCGTGAGAAG | ACATTTGCAGGCGACATCAGC |
| *Wnt9a* | GCAGCAAGTTTGTCAAGGAGTTCC | GCAGGAGCCAGACACACCATG |
| *Wnt10a* | CCTGTTCTTCCTACTGCTGCTGG | CGATCTGGATGCCCTGGATAGC |
| *Wnt10b* | TTCTCTCGGGATTTCTTGGATTC | TGCACTTCCGCTTCAGGTTTTC |
| *Wnt11* | CTGAATCAGACGCAACACTGTAAAC | CTCTCTCCAGGTCAAGCAGGTAG |
| *Wnt16b* | ACTGGATGTGGTTGGGCATC | TAGGCAGCAGGTACGGTTTC |
| *cGas* | AATGATACAGCGCAGCGAGA | CTGCAACAACCCATGCAACA |
| *18S* | CGATAACGAACGAGACTCTG | GACATCTAAGGGCATCACAG |
