# Supplementary figures and images for: Ror2 signaling regulated by differential Wnt proteins determines pathological fate of muscle mesenchymal progenitors
Source: Cell Death Dis. 2024 Oct 29;15(10):784. doi: 10.1038/s41419-024-07173-9 (PMC11519583; doi:10.1038/s41419-024-07173-9)

**
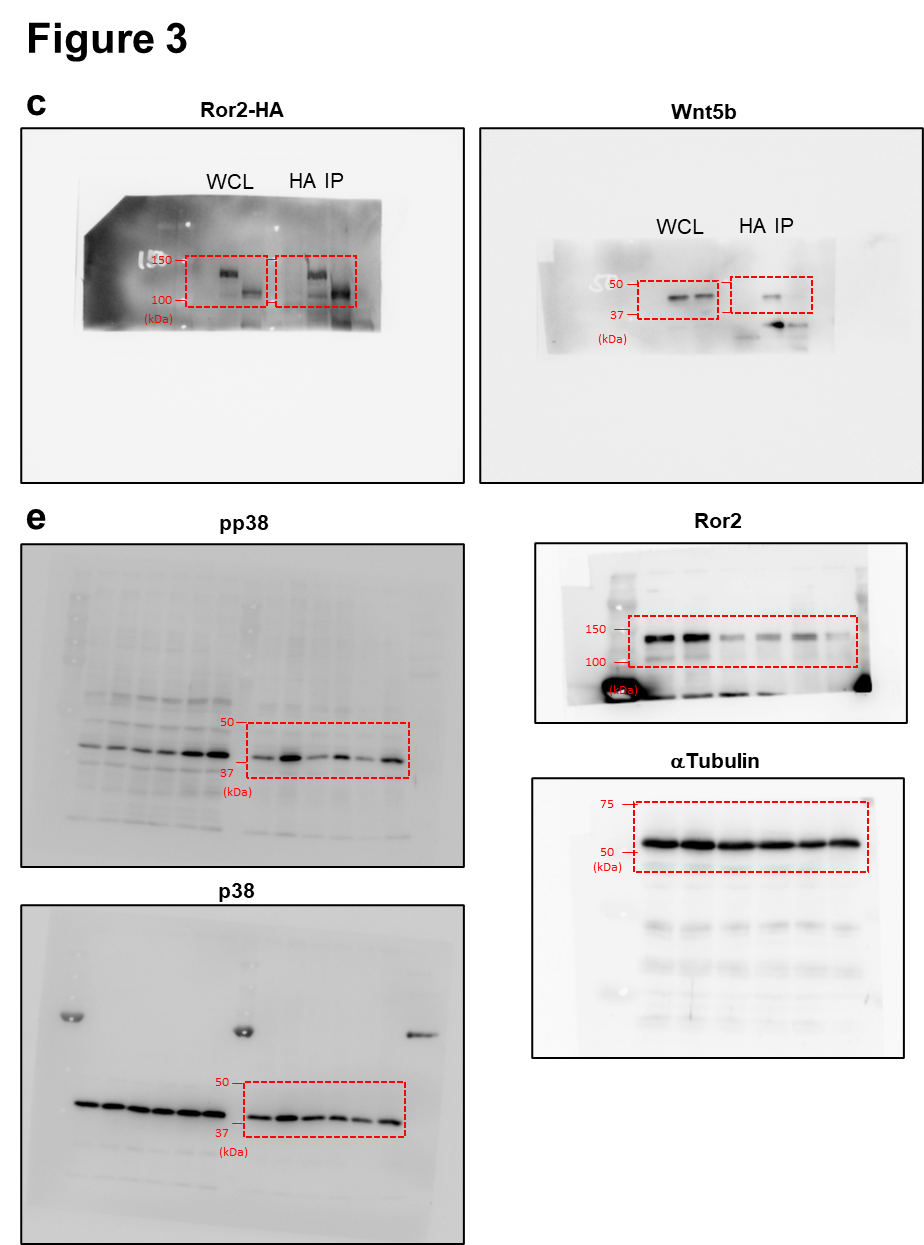
Original western blots**


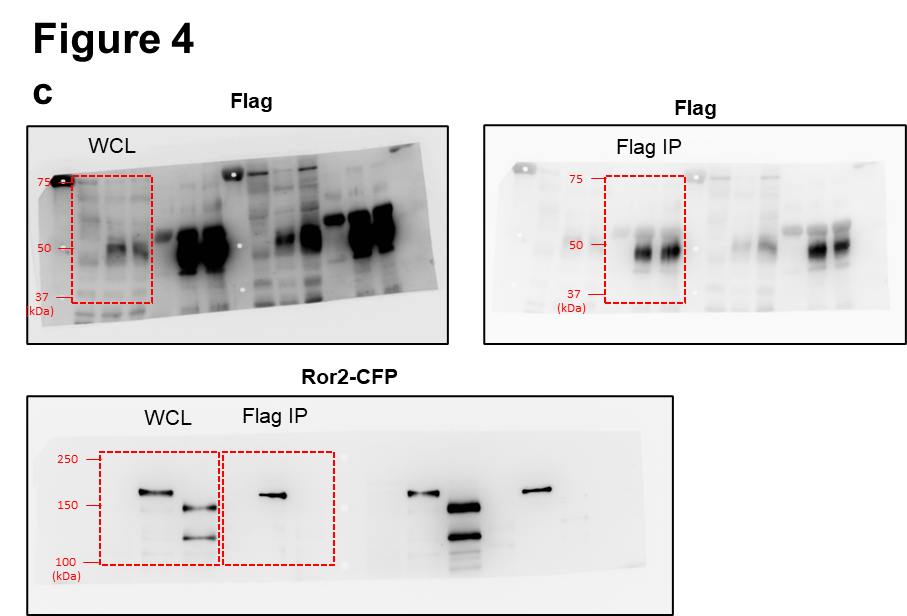


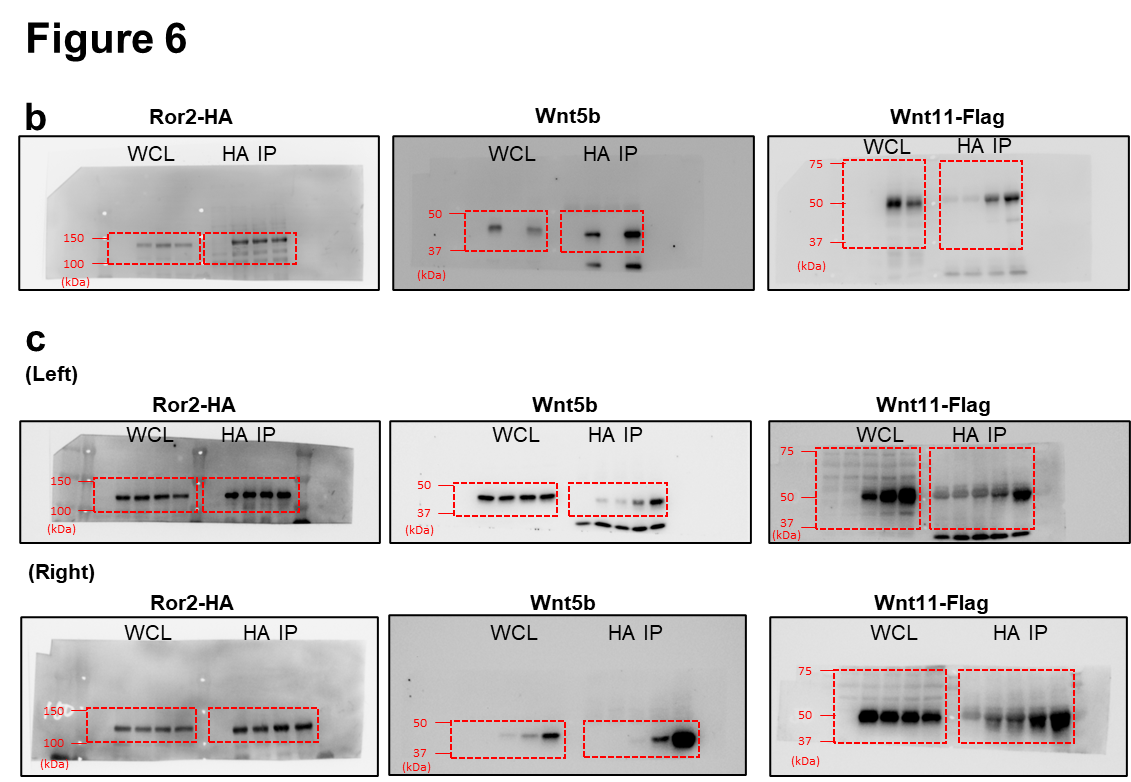


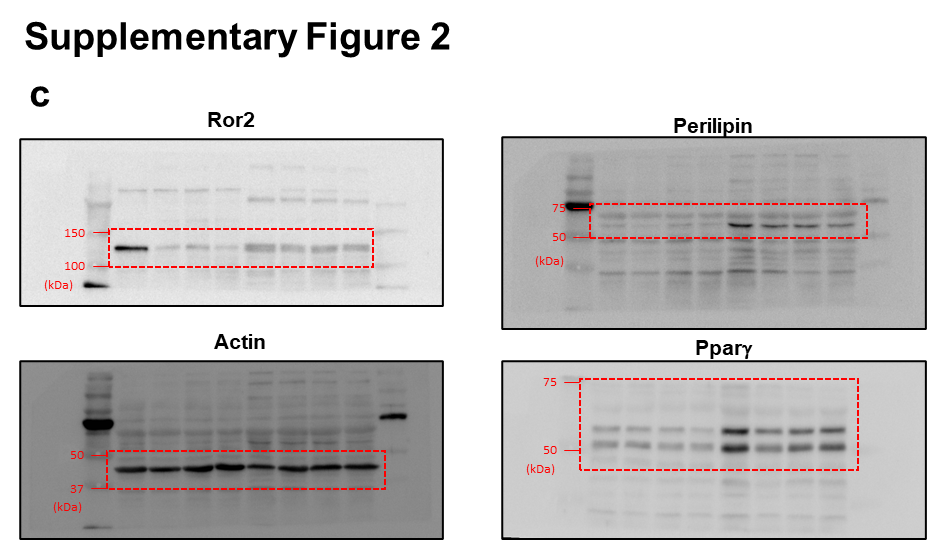


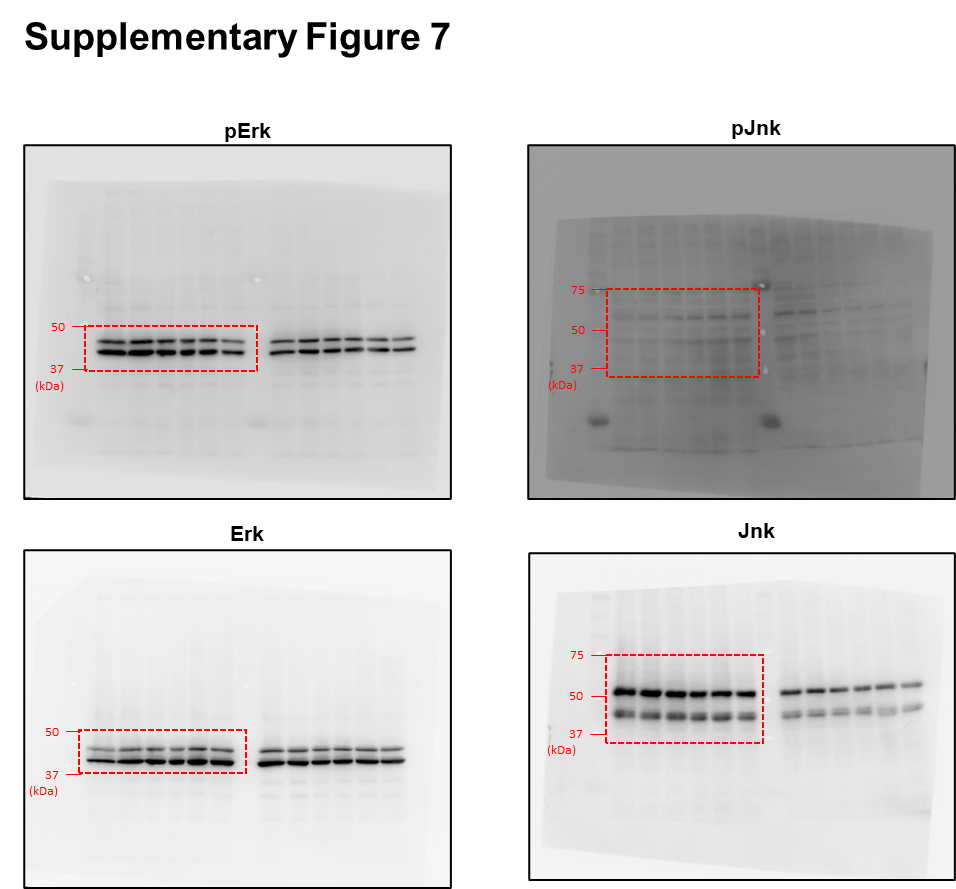


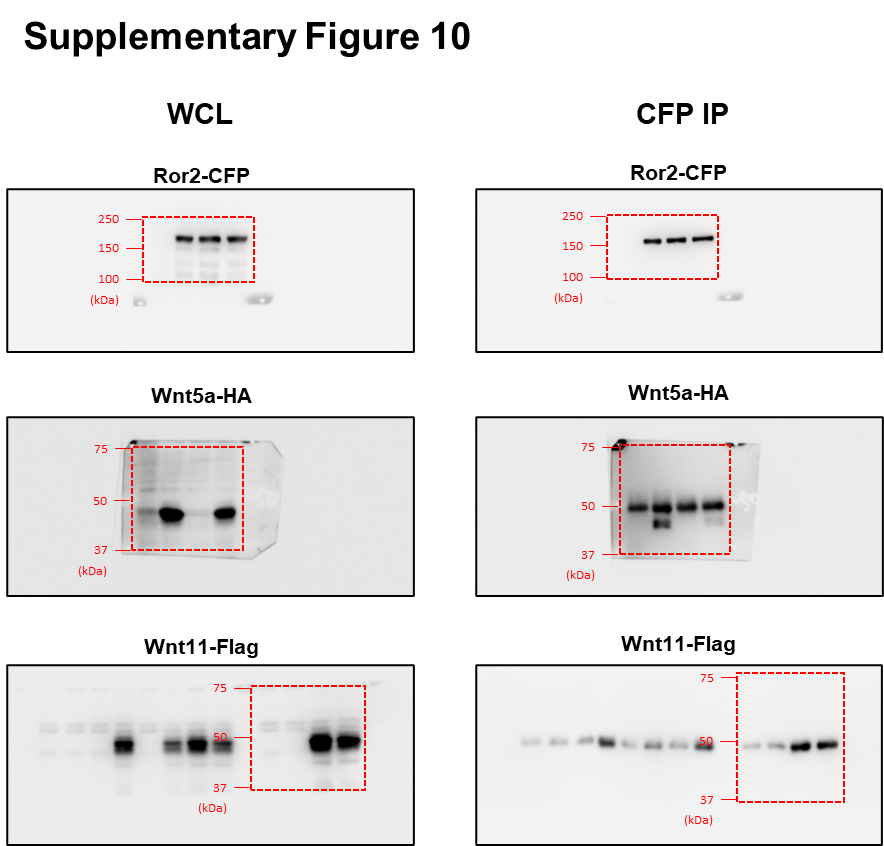

Supplement: Supplementary file 2 — Original western blot [file 41419_2024_7173_MOESM2_ESM.docx]
